# “I Know What I Like” – Indecisiveness Is Unrelated to Behavioral Indicators of Evaluation Difficulties

**DOI:** 10.3389/fpsyg.2021.710880

**Published:** 2021-09-16

**Authors:** Helmut Appel, Birte Englich, Juliane Burghardt

**Affiliations:** ^1^Institute of Applied Social Psychology and Decision Making, University of Cologne, Cologne, Germany; ^2^Division of Clinical Psychology, Karl Landsteiner University of Health Sciences, Krems an der Donau, Austria; ^3^Department of Social Psychology, Universität Hamburg, Hamburg, Germany

**Keywords:** indecisiveness, decision-making, evaluation, explicit and implicit evaluation measurement, evaluative priming, evaluation difficulties, preference uncertainty

## Abstract

Indecisiveness, the subjective inability to make satisfying decisions, is an individual difference trait that may impede effective actions. Mechanisms underlying indecisiveness are largely unknown. In four studies, we tested the prediction that indicators of evaluation difficulty were associated with indecisiveness in simple evaluations. Across studies, indecisiveness was measured via self-report while evaluation difficulties were derived behaviorally from three indicators: difficulty distinguishing between similar evaluation objects (i.e., standard deviation of evaluation ratings), evaluation duration (reaction times), and implicit evaluations (evaluative priming effect) using familiar everyday objects. Study 1 (*N* = 151) was based on attractiveness evaluations of portraits. Studies 2a (*N* = 201) and 2b (*N* = 211) used chocolate as evaluation objects and manipulated to what extent the evaluations were equivalent to a decision. In Study 3 (*N* = 80) evaluations were measured implicitly through evaluative priming using food pictures. Contrary to our predictions, indecisiveness showed no reliable association to any indicator of evaluation difficulty, regardless of type of evaluation object, equivalence of evaluation and decision, and whether evaluation difficulty was based on explicit or implicit evaluations. All null findings were supported by Bayes factors. These counterintuitive results are a first step toward investigating evaluation processes as potential mechanisms underlying indecisiveness, showing that for both explicit and implicit measurements, indecisiveness is not characterized by difficulties when evaluating familiar everyday objects.

## Introduction

In times of countless daily choices, decision problems seem to occur regularly. However, people differ in their general tendency to consider themselves capable or incapable of deciding. This individual difference trait is called indecisiveness (Frost and Shows, 1993). Evaluating, i.e., knowing what one likes and dislikes, is an important requirement for decision making (Heckhausen and Gollwitzer, 1987). Intuitively, evaluation difficulties seem almost synonymous to indecisiveness. The present research, however, supports a clear distinction between indecisiveness and evaluation problems. We present evidence suggesting that important behavioral indicators of evaluation difficulties are unrelated to indecisiveness when evaluating familiar everyday objects.

### Definition of Indecisiveness

Indecisiveness is defined as a dysfunctional personality trait characterized by a generalized difficulty to make decisions (Lauderdale et al., 2019). Being a stable individual difference independent of a particular decision content, it is not to be confused with indecision. Indecision denotes the state of being undecided about a specific decision at hand (Germeijs and De Boeck, 2002). Indecisiveness is an important target of research because it has many problematic correlates. These correlates range from impeded action (Ferrari, 1994), for instance a lack of commitment to academic goals (Germeijs and Verschueren, 2011), to increased risks for mental disorders, such as obsessive-compulsive disorder (e.g., Frost and Shows, 1993), depression (e.g., Leykin and DeRubeis, 2010), or anxiety (Rassin and Muris, 2005a).

Several instruments have been introduced to measure indecisiveness, each with slightly different scopes. For example, the Decision Behaviors Inventory (Barkley-Levenson and Fox, 2016) focuses on indecisiveness-related behaviors. The Indecisiveness Scale by Germeijs and De Boeck (2002) aims to capture various facets of indecisiveness, ranging from behavioral manifestations to decision-making knowledge. The most widely used measure is the Indecisiveness Scale by Frost and Shows (1993) and Lauderdale and Oakes (2021). It has been developed and used in the context of psychological disorders, but also applied to other areas (Rassin, 2007). The scale correlates with related constructs (e.g., an avoidant decision making style, Weinhardt et al., 2012; Bavolar, 2018; certain components of perfectionism, Frost and Shows, 1993; Piotrowski, 2019; or abstract-analytical rumination, Schiena et al., 2013; Piotrowski, 2019), and with symptoms of disorders associated with indecisiveness, especially from the obsessive-compulsive spectrum (Frost and Shows, 1993; Steketee et al., 2003). These findings support its validity. The scale measures indecisiveness based on its cognitive (e.g., worrying), emotional (e.g., fear), and behavioral (e.g., decision delay) aspects. The authors suggest that a concern over mistakes (i.e., making the wrong decision) is the basis of indecisiveness. The scale is thus intended to reflect this conceptualization. The current studies use this definition of indecisiveness.

Referring to Frost and Show’s work, Rassin (2007) proposed a comprehensive model of indecisiveness. It distinguishes between predisposing risk factors, characteristic perceptions of the decision, moderator variables, and typical outcomes. One of the characteristic perceptions of the decision within this model is evaluation difficulty, which is assumed to contribute to indecisiveness. Following the model, our studies look at evaluation difficulties as a phenomenon related to but separate from indecisiveness.

Some model components have been researched extensively and received empirical support. Previous studies focusing on self-report questionnaires have confirmed several of the *predisposing* individual differences specified in the model. These include perfectionism (Gayton et al., 1994; Burgess et al., 2018) and maximizing (Spunt et al., 2009; Barkley-Levenson and Fox, 2016), i.e., the tendency to invest disproportional efforts in finding the best possible decision outcome (Schwartz et al., 2002). Further, the *outcomes* of indecisiveness included in the model have been studied, e.g., worry. In line with this, indecisiveness has been shown to correlate with worry (Rassin et al., 2007; Koerner et al., 2017). The support for longer decision times as a function of indecisiveness is mixed, on the other hand. Some studies show a positive relationship (Frost and Shows, 1993; Patalano and Wengrovitz, 2007). Other findings suggest faster decision times with increasing indecisiveness under some conditions (Barkley-Levenson and Fox, 2016).

Indecisiveness-specific *perceptions* of the decision are another important part of the model. However, cognitive mechanisms shaping these perceptions remain largely unknown. Evaluation difficulties are one of these perceptions. Rassin (2007, p. 1) calls this “valuation difficulty.” According to the model, evaluation difficulties contribute to indecisiveness. This reasoning is in line with other decision making research: Subjective evaluations of choice options are a crucial prerequisite for decisions (Heckhausen and Gollwitzer, 1987). If options cannot be evaluated properly, certainty about preferences cannot be achieved, leaving a necessary condition for choosing unfulfilled. The following studies therefore sought evidence for a relationship between indecisiveness and evaluation difficulties.

### Operationalizing Evaluation Difficulties

Even though the ability to evaluate is a prerequisite for deciding evaluations and decisions are different processes (Montgomery et al., 1994). They produce different and sometimes even conflicting outcomes (Payne et al., 1992). Likewise, indecisiveness clearly goes beyond difficulties in the evaluation of choice objects. For example, emotional components of indecisiveness, like fear of making the wrong choice (Frost and Shows, 1993), can hardly be equated with evaluation difficulties (Rassin, 2007). Thus, an operationalization of evaluation difficulties needs to differentiate indecisiveness from evaluation difficulties.

Since mechanisms leading to evaluation difficulties are not well understood there is no agreed upon operationalization of the concept. To increase the likelihood to find the predicted association between evaluation difficulties and indecisiveness we derived three different operationalizations of evaluation difficulties from the literature. In line with Rassin (2007) (cf. also Germeijs and De Boeck, 2003), we consider the inability to perceive differences between choice options as an important indicator of evaluation difficulties (cf., Anderson, 2003). In decision-making experiments, evaluation difficulty is manipulated by varying the similarity between choice options. Higher similarity leads to decision deferral (Dhar, 1997). A study by Germeijs and De Boeck (2003) supports this reasoning, finding that the impression of equally attractive alternatives predicted career indecision. These findings suggest that indecisiveness-related decision problems may arise because options are perceived too similar, making it hard to distinguish them in terms of preferences. To test this assumption, we measured standard deviations of evaluations as one operationalization of evaluation difficulties.

Another indicator of evaluation difficulties is the time needed for evaluating. Studies manipulating the difficulty of evaluations, for instance through higher ambivalence (Schneider et al., 2015), or higher option similarity (Fiedler and Glöckner, 2012), find longer evaluation times. This is presumably due to increased evaluation difficulty. Also, in simple evaluations, slower evaluations are associated with lower attitude certainty (Tormala et al., 2011). Consequently, indecisiveness may be related to the time needed for evaluating because it reflects evaluation difficulties.

Evaluation standard deviation and evaluation time represent different components of evaluations but can be derived from the same data, so we included both. This also increases the likelihood to capture the hypothesized relationship between indecisiveness and evaluation difficulty. Because both measures are behavior-based and collected without participants’ awareness, they are unsusceptible to the distortions found in self-reports.

Still, they do rely on explicit evaluations. A comprehensive look at the decision-making process, however, also requires implicit measures of evaluations. In contrast to self-report, implicit measures infer evaluations from reaction times. Explicit and implicit measures often diverge when predicting behavior (Kurdi et al., 2019). The source of this divergence is still debated (Corneille and Hütter, 2020). Implicit measures may access different attitudes than explicit measures, for instance, attitudes that might be more difficult to access consciously or that have been acquired in a different way (Corneille and Hütter, 2020). Alternatively, they may measure the same attitude but in a different way, that may for instance be harder to control by the participant (see Gawronski, 2009). Testing the assumed relation between indecisiveness and evaluation difficulties based on both explicit and implicit measures will provide a more complete picture.

### Indecisiveness and Evaluation Difficulty

Although no direct evidence exists, some previous findings suggest a link between indecisiveness and evaluation difficulty. For example, in a study by Rassin and Muris (2005a) indecisiveness correlated with the number of “I don’t know” answers in an opinion survey on controversial topics, which can be interpreted as evaluation difficulties. Another hint can be found in research on rumination. Rumination is defined as “repetitive, prolonged, and recurrent thought about one’s self, one’s concerns and one’s experiences” (Watkins, 2008, p. 163). Experimentally inducing rumination has been demonstrated to result in greater indecisiveness (van Randenborgh et al., 2009). One potential explanation is that the high abstractness of thoughts during rumination blocks access to personal preferences (van Randenborgh et al., 2009). In further support of this interpretation, Schiena et al. (2013) also found that a rumination induction resulted in higher indecisiveness. Importantly, this was true only for abstract-analytical rumination. A concrete-experiential mode of rumination, being more adaptive and focused on problem solving (Watkins, 2008), did not affect decision making.

These findings are in line with evaluation difficulty being an antecedent of indecisiveness, but they provide only indirect evidence. As for implicit measures of evaluations, no empirical research has directly investigated their relationship with indecisiveness so far.

### The Current Research

We therefore tested for associations between evaluation difficulties and indecisiveness in a direct way. We used three behavioral measures that reflect evaluation difficulties: the degree to which objects are evaluated as similar, as shown in the SD of evaluations, the speed of evaluations, and implicit evaluations assessed by evaluative priming.

Study 1 was based on ratings outside the decision context. In Studies 2a and b, we used ratings of potential decision objects and experimentally manipulated the extent to which the rating process was equivalent to an actual decision. We made sure not to confound evaluation difficulties with outcome uncertainty and lack of information – the other cognitive factors contributing to indecisiveness specified in Rassin’s indecisiveness model. Therefore, ratings represented global evaluations of familiar everyday objects. By global evaluations, we refer to evaluations focusing on one highly salient evaluation dimension, i.e., attractiveness of faces in Study 1, and palatability of food in Studies 2a and b. These evaluation objects were chosen because they are ubiquitous and important in people’s everyday experience. Their processing is even associated with specialized cerebral areas (e.g., Jeffreys, 1996; LaBar et al., 2001). Therefore, these stimuli should afford evaluations that can be considered easy and allow for individual difference to exert an influence. For the same reason, we refrained from complex multi-attribute evaluations (e.g., Ferrari and Dovidio, 2000; Baron, 2008). We thus avoided outcome uncertainty and lack of information, the other two indecisiveness-specific perceptions stated in Rassin’s model. Thereby, we can exclude them as rivaling explanations for our results. Studies 1 through 2b relied on explicit evaluations. Study 3, on the other hand, used an evaluative priming paradigm assessing implicit evaluations. Again, evaluation objects were familiar everyday objects (food).

Indecisiveness was operationalized using the Indecisiveness Scale (Frost and Shows, 1993). Originally construed as a unidimensional 15 item measure, several modifications have been proposed. Rassin et al. (2007) introduced a shortened version, excluding four items because they reflect indecision in specific decisions instead of general indecisiveness. Also, different factor structures have been suggested (e.g., Patalano and Wengrovitz, 2006; Rassin et al., 2007; Spunt et al., 2009). Recently, a comprehensive factor analysis derived two sub-dimensions of the scale: Aversive Indecisiveness and Positive Attitudes Toward Decision-Making (Lauderdale and Oakes, 2021). Aversive Indecisiveness primarily encompasses anticipation of negative decision-making outcomes (Spunt et al., 2009; Lauderdale and Oakes, 2021). It is therefore closer to the original definition of indecisiveness. In contrast, the Positive Beliefs Toward Decision-Making items might measure a different construct. Lauderdale and Oakes (2021) suggest they capture decisional self-efficacy, rather than indecisiveness. As there is no definite certainty about the factor structure to date, we used the original Indecisiveness Scale (Frost and Shows, 1993), treating it as unidimensional. However, we also repeated all main analyses using the shortened scale proposed by Rassin et al. (2007), and the Aversive Indecisiveness subscale presented by Lauderdale and Oakes (2021). Conclusions drawn from our data remained unchanged. The results are presented in the respective section “Additional Analyses,” and details are given in Supplementary Material.

## Study 1: Overview and Hypotheses

Study 1 tested the hypothesized evaluation difficulties related to indecisiveness by having participants rate the overall attractiveness of faces sequentially. We expected indecisiveness to correlate negatively with the standard deviation of ratings and positively with duration of ratings.

### Method

#### Participants

Advertised as a study on attractiveness, we collected data from 151 United States-based participants via MTurk (45.0% female, *M*_age_ = 35.75 years, *SD* = 12.31). They received $0.45 for compensation. To determine the resulting power, we used the effect size of *r* = 0.31 based on the correlation between indecisiveness and another behavioral measure reported by Rassin and Muris (2005b). The resulting power estimate was β = 0.97 (G^∗^Power, Faul et al., 2007).

#### Evaluation Objects

All materials and instructions of this and the following studies can be found in the Supplementary Material (ESM 1.1, 2a.1, 2b.1, and 3.1). We used the 20 most attractive portraits of both sexes from Corneille et al. (2005) as evaluation objects. The portraits were black and white photographs of only the depicted person’s head and upper body stemming from an online casting database. The authors took care to standardize and pretest the stimuli (Corneille et al., 2005). This allowed us to draw on the existing attractiveness ratings and rely on the highly standardized picture composition.

#### Procedure and Materials

The informed consent and introduction were followed by demographic questions. These included a question about sexual attraction (1 = *clearly more to men*, 2 = *slightly more to men*, 3 = *slightly more to women*, 4 = *clearly more to women*). Faces of the preferred sex were presented first. Pictures were randomized within one sex. Participants rated the attractiveness of each picture on three items (e.g., “How attractive do you personally think this face is?” α_minimum_ = 0.91) using continuous sliders (range: 1 = *not at all* to 49 = *extremely*). The similarity of the ratings was determined by the standard deviation across all ratings. A low rating standard deviation implied perceived similarity of options and thus evaluation difficulties.

Rating duration was used as second indicator: longer rating times indicated evaluation difficulties. Afterwards, participants reported their trait indecisiveness on the original 15-item version of the Indecisiveness Scale (Frost and Shows, 1993). The scale has excellent internal reliability (α = 0.91, Frost and Shows, 1993, Study 1). Sample items include: “I often worry about making the wrong decision,” and “I find it easy to make decisions” (reverse coded). Participants gave their answers on a 5-point Likert scale (1 = *strongly disagree* to 5 = *strongly agree*). Internal reliability in the current sample was α = 0.91. Finally, they reported additional demographic information, assumptions about the purpose of the study, and their distraction during participation on an 11-point scale (1 = *not at all* to 11 = *very much*). Participants were offered debriefing.

### Results

Correlations between non-normally distributed variables are reported using Kendall’s tau. Rating times 3 SD above or below the individual mean were excluded as outliers (2.2%; cf. Koop, 2013). Average indecisiveness was 2.47 (*SD* = 0.74), average rating standard deviation was 10.23 (*SD* = 4.04), and average rating time was 9.25 s (*SD* = 3.77 s) after correcting for outliers. Rating standard deviation and rating time were uncorrelated, τ = −0.03, *p* = 0.59. We therefore analyzed the two indicators of evaluation difficulty separately with Bonferroni corrected *p-*values for multiple testing. Indecisiveness did not correlate with rating standard deviation, τ = −0.004, *p* = 1, CI_95%_ [−0.11, 0.12]. Because a negative result does not provide evidence for the null hypothesis in classical null hypothesis significance testing (NHST, e.g., Cohen, 1994), we conducted a Bayesian analysis. The Bayes factors quantify the odds for the observed data given the respective hypothesis compared to the alternative hypothesis (Wagenmakers, 2007). Bayes factors between 3 and 10 indicate substantial evidence, while factors beyond 10 indicate strong evidence in favor of the respective hypothesis. The Bayes factor for the null hypothesis (BF_01_) of 9.37 suggested that the results substantially favored no correlation between indecisiveness and rating standard deviation. Rating time did correlate with indecisiveness, but contrary to the hypothesis the correlation was negative, τ = −0.17, *p* = 0.004, CI_95%_ = [−0.27, −0.06], with BF_10_ = 12.40 favoring the alternative hypothesis.

### Additional Analyses

To scrutinize the results, we excluded distracted participants and limited analyses to photos of the preferred sex. We also used the shortened 11-item version of the Indecisiveness Scale by Rassin et al. (2007, hereinafter referred to as IS-Short) excluding all situation-specific items (e.g., “When ordering from a menu, I usually find it difficult to decide what to get”), and the Aversive Indecisiveness subscale identified by Lauderdale and Oakes (2021, hereinafter referred to as IS-AI). The unpredicted negative correlation between indecisiveness and rating time disappeared when considering the preferred sex only, and it was not significant after Bonferroni-correction for the IS-Short. Otherwise, the results did not change. Details are given in ESM 1.2.

### Discussion

Study 1 tested whether indicators of evaluation difficulty correlated with indecisiveness in global evaluations of familiar everyday stimuli. Contrary to our hypothesis, no correlation was found when looking at the similarity of ratings. When looking at rating duration, the correlation was even negative, indicating that indecisiveness was associated with faster instead of slower evaluations. Although this was contrary to our prediction, it might reflect indecisiveness-related evaluation avoidance. If indecisiveness is correlated with evaluation difficulty, faster evaluation times might actually reflect avoidance of the evaluation.

This reasoning is in line with counterintuitive findings suggesting a negative relationship between indecisiveness and the time needed for decision-related processes. Specifically, Barkley-Levenson and Fox (2016) surprisingly found a correlation between indecisiveness and impulsivity. They interpreted this result as a motivation to quickly end the unpleasant experience of a decision. This idea is further supported by research on intolerance of uncertainty. Intolerance of uncertainty is a personality trait characterized by “[negative] cognitive, emotional, and behavioral reactions to uncertainty” (Freeston et al., 1994, p. 792). It is strongly correlated with indecisiveness (e.g., Rassin et al., 2007; Koerner et al., 2017) and thought to be one of its antecedents (Rassin, 2007). Luhmann et al. (2011) showed that intolerance of uncertainty was associated with choosing a quickly available reward, even if this reward was less probable and less valuable than a delayed reward. This choice was likely to avoid the uncertainty during the delay. In a similar way, indecisiveness might also promote faster decisions under some circumstances. This again, might extend to indecisiveness and evaluations, with faster evaluations reflecting evaluation avoidance. However, the negative correlation of indecisiveness and evaluation time in Study 1 was very small. Also, it depended on which sex was rated and which version of the Indecisiveness Scale was looked at. It should therefore be interpreted cautiously and tested again in the following studies. Overall, the results suggest that indecisiveness is not related to difficulties evaluating familiar objects *per se*.

The stimuli used do not represent actual decision objects. This was done in order to separate evaluation and decision processes, which, despite frequently occurring together (Baron, 2008), are characterized by different cognitive processes (Montgomery et al., 1994). However, it is possible that indecisiveness only involves evaluation difficulties if the evaluations are relevant for decision-making. Previous research by van Harreveld et al. (2009) is in line with this reasoning. They showed that ambivalence about which option to prefer leads to uncertainty and negative affect only if a decision must be made. Our additional analyses using only pictures of the preferred sex contradict this idea: Although these pictures can be seen as “decision options,” for instance as romantic or sexual partners (DeBruine, 2004), they did not produce a correlation between indecisiveness and evaluation difficulty. However, this interpretation is speculative. We therefore used clearly decision-related evaluation objects in Studies 2a and b.

If indecisiveness only correlates with evaluation difficulties for objects with decision relevance, the question arises how closely the evaluations have to resemble a true decision in order for that to happen. This question is explored in the following Studies 2a and 2b.

## Study 2A: Overview and Hypotheses

In Study 2a, we tested whether indecisiveness is associated with difficulties in evaluations when they are more equivalent to a decision. To manipulate the equivalence of evaluations and decisions we systematically modified the evaluation process to approximate a decision-making process. We used chocolate bars as evaluation objects, which simultaneously were decision objects because participants could choose a chocolate bar as a reward for participation. This allowed us to manipulate two aspects to make the evaluations resemble a decision more closely. First, we varied whether options were rated one at a time (sequential rating), or whether two options were being rated at once (simultaneous rating). In simultaneous ratings direct comparisons between options are highlighted, so the focus is on *preferring* one option over the others (Hsee and Leclerc, 1998). They are thus more similar to a decision than sequential ratings. The difference between sequential and simultaneous rating also affects the evaluation itself (e.g., Bazerman et al., 1992). It is thus possible that evaluation difficulty only occurs in indecisiveness when several options are rated simultaneously.

Second, we varied whether the ratings had direct influence on the choice. Participants were either informed that they would receive their highest rated chocolate bar at the end (consequence of ratings), or that the ratings were independent of their choice (no consequence of ratings). Evaluations which directly determine the reward are obviously more similar to a decision than evaluations independent of the reward choice. At the same time, evaluations and preferences change depending on whether they are viewed as pure evaluations or decisions (Payne et al., 1992; Montgomery et al., 1994).

Arguably, a participant rating several items at once (simultaneous evaluation) while being aware that they will receive the highest rated option (consequence of rating) logically makes a decision. Either of these two factors or a combination of both could be a necessary condition for an association between indecisiveness and evaluation difficulty. Building on this reasoning, we predicted that the correlation between indecisiveness and evaluation difficulty increases when two options are rated simultaneously and when the choice is determined by the ratings. The correlation should be strongest when both factors are combined.

### Method

#### Participants

For the study 205 people participated after being approached on campus at a German university. The alleged purpose of the lab-study was to pre-test chocolate bars for future studies. The data from four participants were not successfully recorded. Accordingly, the final sample size was 201 (85.1% female, *M*_age_ = 23.6 years, *SD* = 3.69). Using the same assumptions as Study 1, estimated power was β = 0.94. The study had a 2 (presentation of the options: sequential vs. simultaneous) by 2 [consequence of the ratings: choice dependent on rating (“with consequence”) vs. choice independent of rating (“without consequence”)] experimental design. Participants were randomly assigned to conditions.

#### Procedure and Materials

**FIGURE 1:**
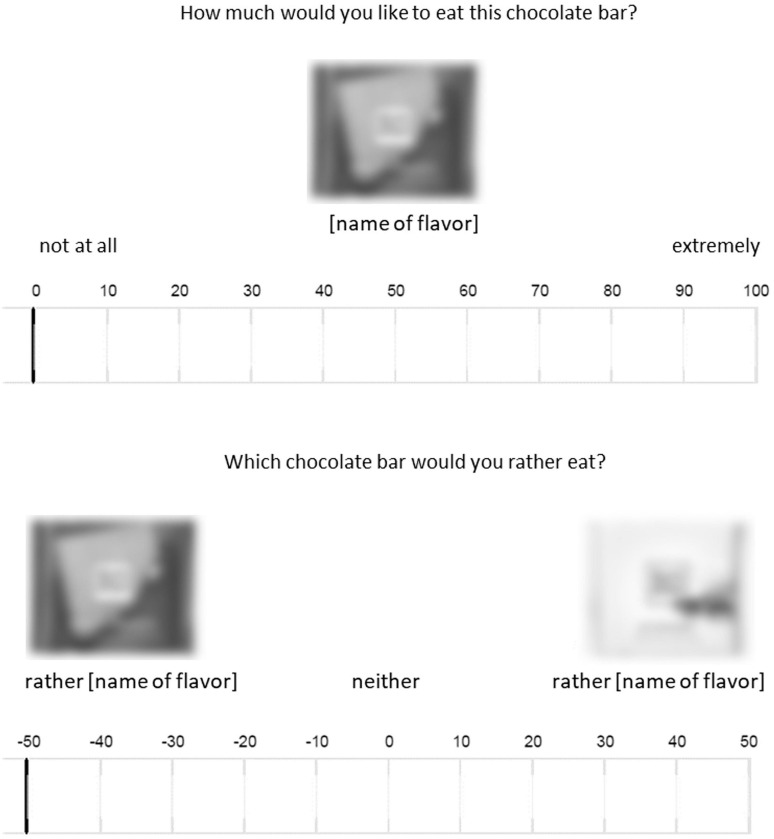
Example of a rating trial in the sequential rating condition (top) and the simultaneous rating condition (bottom) in Study 2a.

Up to three individuals participated simultaneously on computers in individual cubicles. The rating task followed the informed consent. Eight easily comparable and distinguishable flavors of a well-known chocolate brand (Ritter Sport©) served as evaluation objects. Participants in the condition with consequence were informed that they would receive their highest rated chocolate bar as a reward, or one of the higher rated chocolate bars in the condition with simultaneous ratings. In the condition without consequence, however, it was made clear that the ratings had no influence on the reward. Further, the instructions included an attention check in the form of an instructional manipulation check (IMC, Oppenheimer et al., 2009). This was followed by the rating of the chocolate bars. In the condition with sequential presentation the different flavors were rated separately in a randomized order. Participants saw a picture of the respective flavor and indicated how much they would like to eat it on a continuous slider scale ranging from 0 (*not at all*) to 100 (*extremely*). In the condition with simultaneous ratings two flavors were presented per evaluation. Flavors displayed together had been rated similarly in an online pre-test (*n* = 71) to allow for high difficulty. Participants rated which flavor they would prefer to eat using a slider (−50 to 50) with a picture of one of the two chocolate bars at each end of the scale (0 = *neither*). We randomized the order in which the pairs were presented, and which flavor was shown on which side. Figure 1 shows the setup for both conditions.

Like in Study 1, rating time was used as an indicator for evaluation difficulties in addition to rating standard deviation. We used the sum of all rating times instead of the mean to minimize rating time differences due to the sequential vs. simultaneous ratings. After the rating, participants chose a chocolate bar as a reward. Participants in the condition without consequence chose from a second, previously unseen set, while participants in the condition with consequence were presented with the set of chocolates they had already rated. For exploratory reasons, we asked participants how difficult they perceived the choice using six items and recorded their decision time.

Afterwards, participants filled in the Indecisiveness Scale (α = 0.89), followed by demographic questions. To check whether participants remembered the consequence of rating manipulation, participants indicated whether their ratings were announced to have consequences or not (manipulation check). Finally, all participants were offered a debriefing.

### Results

#### Descriptive

Like in Study 1 we excluded rating times 3 *SD* above the mean of their respective conditions (1.2%). The mean for indecisiveness was 2.68 (*SD* = 0.70), the mean standard deviation of the ratings was 28.04 (*SD* = 11.13), and the mean total rating time was 35.95 s (*SD* = 10.57 s). As in Study 1, the two indicators of evaluation difficulty, i.e., rating standard deviation and rating time, did not correlate, *r* = 0.13, *p* = 0.06, so we analyzed them separately.

#### Manipulation Check

The manipulation check showed that 69 participants (34.3%) failed to recall whether their ratings were announced to have consequences or not. Excluding participants who failed the manipulation check did not impact the results. The same was true for excluding the 72 participants (35.8%) who failed the IMC (see section “Additional Analyses”).

#### Association Between Indecisiveness and Evaluation Difficulties

We expected the association between indecisiveness and evaluation difficulty to increase when options were presented simultaneously and when the choice depended on the ratings. We included indecisiveness (centered), the two factors (consequence of the ratings and presentation of the options, dummy-coded), all two-way interaction terms, and the three-way interaction in a linear regression model as predictors of the rating standard deviation. Table 1 shows the results. While the model accounted for a substantial portion of the total variance, this was mainly due to the sequential presentation resulting in a significantly lower standard deviation, which merely reflects the different modes of presentation. Importantly, the expected interactions between indecisiveness and both factors, as well as their three-way interaction could not be confirmed.

**TABLE 1:** Regression of evaluation difficulty (rating standard deviation and rating time) on indecisiveness, consequence of ratings, presentation, and their interactions in Study 2a.

	Rating standard deviation				Rating time (s)			
	*b*	*t*	BF_01_	CI_95%_	*b*	*t*	BF_01_	CI_95%_
Indec.	0.28	0.06	3.00	[−10.38, 9.90]	7.01	1.65	1.06	[−1.81, 16.48]
Cons.	−0.21	0.10	2.99	[−4.78, 4.30]	–1.21	0.63	3.10	[−5.05, 2.50]
Pres.	−6.64	3.08^**^	0.05	[−10.85, −2.70]	8.24	4.34^***^	<0.001	[4.47, 11.84]
Indec. × Cons.	−8.46	1.02	1.88	[−26.20, 8.74]	–7.29	1.00	2.34	[−23.42, 7.49]
Indec. × Pres.	−1.57	0.20	2.95	[−14.81, 13.89]	–3.26	0.46	3.37	[−16.60, 10.01]
Cons. × Pres.	−1.00	0.33	2.86	[−7.35, 5.43]	3.31	1.23	1.84	[−2.03, 8.87]
Indec. × Cons. × Pres.	6.70	0.57	2.60	[−15.33, 27.99]	7.15	0.69	2.99	[−14.75, 28.91]
Full model	*R* ^2^	0.12				0.36		
	*F*	3.89^**^				15.74^***^		

*Indec., indecisiveness; Cons., consequence of the ratings (0 = without consequence, 1 = with consequence); Pres., presentation of options (0 = simultaneously, 1 = sequentially); BF_01_, Bayes factor for null hypothesis; coefficients are unstandardized; indecisiveness scores were log-transformed due to deviations from normal distribution; *p*-values Bonferroni-corrected for the number of dependent variables.*

^**^p < 0.01, ***p < 0.001.

We calculated the same model using rating time as the criterion (Table 1). This resulted in the same pattern as for rating standard deviation. Here too, the only significant effect was the sequential presentation leading to faster ratings. The Bayes factors supported the null hypotheses for all effects pertaining to indecisiveness (Table 1).

### Additional Analyses

Like in Study 1 we conducted additional analyses to test the robustness of the results. We excluded participants who had failed the IMC and the manipulation check. We repeated our main analysis using the IS-Short and the IS-AI. Details are found in ESM 2a.2. Results remained unchanged.

### Discussion

In Study 2a we created conditions that either corresponded to a mere evaluation or were equivalent to a decision, assuming that indecisiveness would be associated to evaluation difficulties if ratings resembled a decision (cf. van Harreveld et al., 2009). Decisions are characterized by simultaneous rather than sequential ratings of options (Bazerman et al., 1992) and usually the decision is determined by the evaluation of options relative to one another (Payne et al., 1992). Depending on condition, participants made evaluations in which several items were evaluated simultaneously and in which the evaluation determined which item participants received. Yet even under these conditions, we found no correlation between indecisiveness and either indicator of evaluation difficulty. Thus, we replicated and extended the findings of Study 1.

It should be noted that the simultaneous and sequential rating conditions in Study 2a were not strictly comparable. The simultaneous rating was relative, i.e., participants had to express a *preference* (“Which chocolate bar would you rather eat?”), while the sequential ratings were absolute (“How much would you like to eat this chocolate?”). Mixing of relative and absolute ratings in Study 2a can be seen as a confound. In Study 2b, we therefore implemented simultaneous ratings which retained an individual rating for each option. That is, we adapted our paradigm to avoid the confounding between simultaneous and relative ratings.

## Study 2B: Overview and Hypotheses

Although Study 2b constituted a replication of Study 2a, an important modification was made. In the condition with simultaneous presentation participants still rated two flavors at a time and in comparison to one another. However, each flavor was assigned its own rating (Bouyssou et al., 2011). As in Study 2a we expected the simultaneous presentation of the objects and the consequence of the ratings for the choice to increase the correlation between indecisiveness and evaluation difficulty, and the correlation to be strongest for a combination of those two factors.

### Method

#### Participants

Participants were approached on campus at a German University and 211 people agreed to participate (82.0% female, *M*_a__ge_ = 23.13 years, *SD* = 4.16). They were randomly assigned to the four conditions of the 2 (presentation: sequential vs. simultaneous) by 2 (consequence of the ratings: with vs. without consequence) experimental design.

#### Procedure and Materials

**FIGURE 2:**
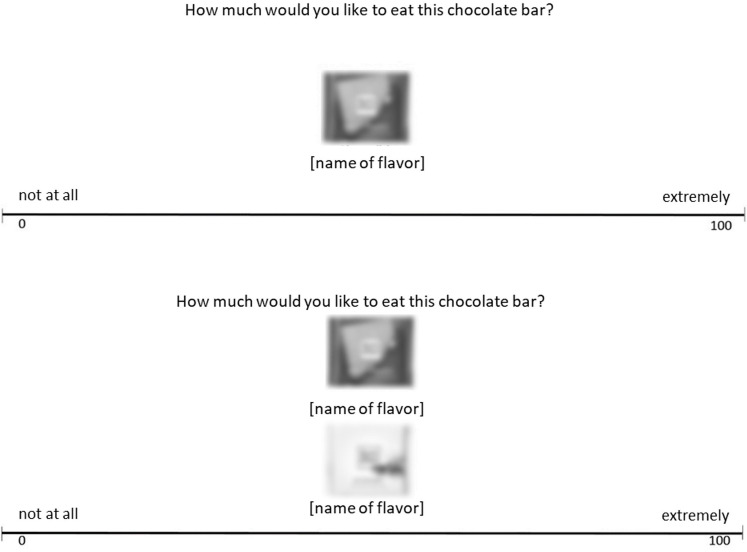
Example of a rating trial in the sequential rating condition (top) and the simultaneous rating condition (bottom) in Study 2b. Pictures of each chocolate flavor could be dragged and dropped onto the rating scale individually.

Procedure and materials largely matched Study 2a. However, in the simultaneous presentation condition, two flavors were rated in comparison to each other, yet both flavors received an individual rating. Participants dragged and dropped the pictures of the chocolates onto the rating scale (Hout et al., 2013; Koch et al., 2016). This enabled us to use the same rating scale in the simultaneous and the sequential condition, with the only difference being the number of flavors rated per round (one in the sequential and two in the simultaneous rating condition). The scale extended horizontally across the screen and ranged from 1 (*not at all*) on the left side to 100 (*extremely*) on the right (see Figure 2). In order to become familiar with the scale, participants practiced with placeholder pictures first. This also served to help internalize the instructions despite the large amount of text. The ratings corresponded to the horizontal position to which the picture was dragged, measured in pixels (1–1680).

### Results

#### Descriptive

Rating times 3 *SD* above or below the mean of the respective condition were excluded from the analysis (1.2%, cf. Studies 1 and 2a). The mean was 2.74 (*SD* = 0.63) for indecisiveness, 388.94 px (*SD* = 117.64 px) for the rating standard deviation and 41.76 s (*SD* = 10.70 s) for the totaled rating time. As in Studies 1 and 2a the indicators of evaluation difficulty, i.e., rating standard deviation and rating time, did not correlate, *r* = −0.10, *p* = 0.17, so we analyzed them separately.

#### Manipulation Check

According to the manipulation check, 62 participants (29.4%) did not remember which consequence their ratings were supposed to have. Excluding participants who failed the manipulation check did not impact the results, as was the case with the ICM (24.6% failure, see section “Additional Analyses”).

#### Correlation Between Indecisiveness and Evaluation Difficulty

In order to test whether the experimental conditions moderated the correlation between indecisiveness and evaluation difficulty, we regressed the similarity (i.e., standard deviation) of the ratings on indecisiveness (centered), the two experimental factors, consequence and presentation (dummy-coded) and all two- and three-way interaction terms. Table 2 summarizes the results. In contrast to Study 2a, in which the mode of presentation influenced the rating standard deviation and the rating time, no such effect could be found here. The Bayes factors indicated no existing effects for indecisiveness, nor any interaction terms including indecisiveness (see Table 2).

**TABLE 2:** Regression of evaluation difficulty (rating standard deviation and rating time) on indecisiveness, consequence of ratings, presentation, and their interactions in Study 2b.

	Rating standard deviation				Rating time (s)			
	*b*	*t*	BF_01_	CI_95%_	*b*	*t*	BF_01_	CI_95%_
Indec.	−13.28	0.46	2.27	[−69.13, 46.12]	0.30	0.11	2.55	[−5.57, 6.58]
Cons.	6.23	0.27	2.41	[−37.88, 54.34]	–2.12	1.02	1.64	[−6.52, 2.50]
Pres.	22.74	0.97	1.67	[−19.49, 64.79]	0.87	0.41	2.39	[4.47, 11.84]
Indec. × Cons.	5.53	0.15	2.46	[−73.06, 84.34]	0.29	0.09	2.56	[−23.42, 7.49]
Indec. × Pres.	19.73	0.53	2.20	[−54.21, 93.46]	2.89	0.87	1.86	[−16.60, 10.01]
Cons. × Pres.	−32.76	1.00	1.63	[−97.13, 31.71]	3.26	1.10	1.53	[−2.03, 8.87]
Indec. × Cons. × Pres.	−22.15	0.42	2.31	[−129.34, 82.52]	–0.23	0.05	2.57	[−14.75, 28.91]
Full model	*R* ^2^	0.01				0.03		
	*F*	0.29				0.83		

*Indec., indecisiveness; Cons., consequence of the ratings (0 = without consequence, 1 = with consequence); Pres., presentation of options (0 = simultaneously, 1 = sequentially); BF_01_, Bayes factor for null hypothesis; coefficients are unstandardized; *p*-values Bonferroni-corrected for the number of dependent variables.*

### Additional Analyses

We conducted the same additional analyses as in Study 2a. Details are found in the ESM 2b.2. Excluding participants who failed the attention or manipulation check did not impact the results, nor did using the IS-Short and the IS-AI in the main analysis.

### Discussion

In line with Study 2a, we found no correlation between indecisiveness and evaluation difficulty in Study 2b, regardless of the similarity between evaluating and deciding. As a near-identical replication of Study 2a, Study 2b was aimed at overcoming the confounding factor created by the relative ratings of two chocolate flavors when presented simultaneously. Yet, the high rate of failed attention checks is a limitation, as it indicates that many participants were not aware of the consequence (or lack thereof) of their ratings. Even though the instructions were illustrated using pictures and, in Study 2b, practice opportunity, the relatively large amount of text could have contributed to this problem. However, excluding these cases did not alter results.

To summarize, no correlation emerged between indecisiveness and evaluation difficulties across three studies, regardless of how similar the rating process was to making a decision. The three previous studies measured evaluation difficulties based on explicit measures. An alternative to explicit measures are implicit measures, which often provide divergent results from explicit measures (Corneille and Hütter, 2020). In Study 3, we explore whether the findings in explicit and implicit measures converged.

## Study 3: Overview and Hypotheses

Study 3 tested the assumption that indecisiveness is related to evaluation difficulties using evaluative priming, an established implicit measure (Wittenbrink, 2007). Since implicit and explicit measures often diverge, we tested whether an indicator of evaluation difficulty correlated with indecisiveness when measured by implicit measures, even though this association was absent for explicit measures. In the evaluative priming paradigm, participants have to categorize a target as positive or negative as quickly as possible. The target is preceded by a prime (i.e., evaluation object). The prime has a valence that is congruent or incongruent to the target valence. Responses in congruent trials are typically faster and more accurate than in incongruent trial, which is termed the evaluative priming effect. The evaluative priming effect increases for more extreme evaluation objects (Giner-Sorolla et al., 1999). To sum up, we assumed that indecisiveness involves less clear (i.e., less extreme) evaluations. Therefore, we predicted that implicit evaluations of objects should be weaker with increasing indecisiveness, producing a reduced evaluative priming effect.

### Method

#### Participants and Design

We recruited participants on campus at a German university. The experiment was described as a reaction time task. Participants received candy as reward. Data of one participant was not successfully recorded. The final sample consisted of 80 participants (85.0% female, *M*_a__ge_ = 22.8 years, *SD* = 4.14). Assuming the same effect size as in the previous studies power was β = 0.81.

#### Priming Stimuli

In order to ensure that the material was relevant to real life decisions, we used food items as primes, which are common choice objects and established primes (Lamote et al., 2004). We selected three positive food pictures from the database food.pics (Blechert et al., 2014) as primes. We included neutral primes to create a baseline (see Wentura, 1999). Contrary to our expectations, however, neutral primes behaved like positive primes. That is, they produced faster reaction times for positive target stimuli, even though they were only rated medium in the *explicit* evaluation. They could therefore not be used to establish a baseline (see Wentura, 1999) and were dropped from the analysis.

In order to ensure recognizability despite short presentation times, we only used pictures with recognition ratings near the maximum. However, there were no distinctly negative foods with high recognizability in the available databases. Thus, we carefully researched new stimuli for this category. All primes were presented as 575 × 300-px pictures on a white background.

We used 20 positive and 20 negative German nouns from the Berlin Affective Word List – Reloaded (Võ et al., 2009) as targets. According to the norms, the emotional valence was higher for positive (*M* = 2.27) than for negative words (*M* = −2.31), *t*(38) = 62.16, *p* < 0.001. We only used words with six to eight letters and exactly two syllables in order to keep processing effort constant.

#### Procedure and Materials

Participants took part in individual cubicles wearing soundproof headphones. The priming task was controlled by DirectRT, the rest was programmed using Unipark. After giving consent, participants first performed the evaluative priming task. They were informed that they were to categorize the target words as positive or negative, by pressing the left mouse button with the left index-finger for positive words and the right mouse button with the right index-finger for negative words as fast and accurately as possible. Further, they were informed that a picture would be shown briefly before every word, which they should pay attention to while only reacting to the words.

**FIGURE 3:**
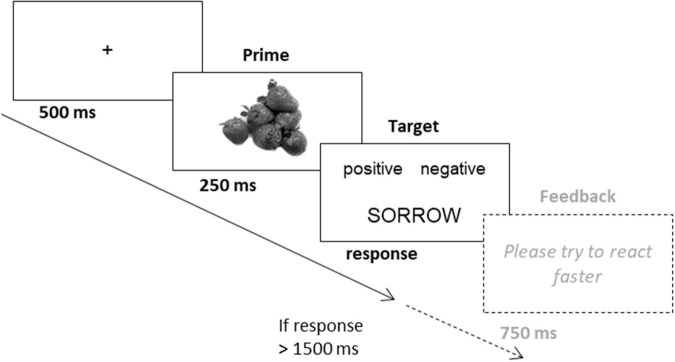
Example of an incongruent trial with a positive prime and a negative target in the evaluative priming task in Study 3.

Overall, participants were presented with three blocks of 126 trials each, preceded by 18 practice trials. Trials were separated by a white screen for 2000 ms. Blocks were separated by 30 s breaks. Positive and negative pictures (primes) followed by a positive or negative word (target) each made up 1/6 of the trials, while the remaining trials contained neutral primes. This resulted in 63 trials each with a positive or negative prime followed by a congruent and incongruent target, respectively. Presentation order as well as the combinations (congruence of prime and target valence) of the stimuli was randomized across all three blocks. Thus, each of the nine prime stimuli was presented 42 times, 21 times followed by a positive and a negative word, respectively. The frequency of the target stimuli was not controlled (randomized presentation with replacement), as we did not expect any target-specific effects. A sample trial can be seen in Figure 3. The priming task took about 25 min.

Afterwards participants completed the Indecisiveness Scale (α = 0.89). Then, they were asked about their current appetite on three 7-point Likert scales (e.g., “How hungry are you at this moment?” 1 = *not at all*, 7 = *very much*). The three items showed excellent internal consistency (minimum α = 0.94), so we combined them. Subsequently, we asked participants to rate the valence of all prime stimuli in random order with an explicit measure consisting of three items (e.g., “How much would you personally like to eat this food?” from 1 = *not at all*, 9 = *very much*, α = 0.81–0.92). The three items were combined for each prime stimulus. Further we inquired about the recognizability and the familiarity of each stimulus using two 7-point Likert scales (1 = *not at all*, 7 = *very much*). Participants were asked whether they had seen each prime stimulus during the reaction time task (*yes*, *no*, or *unsure*). Means for valence, recognizability, and familiarity can be seen in Table 3. Valence ratings produced the expected pattern in that positive primes were rated more positively than negative stimuli. Recognizability and familiarity were lower for negative than for positive stimuli. Still, only in 3.3% of cases did participants report not having seen or being unsure of having seen a negative stimulus during the task. Thus, overall recognizability was high. Demographic questions concluded the study. Afterwards participants received their reward and the opportunity for debriefing.

**TABLE 3:** Valence, recognizability, and familiarity ratings of positive and negative prime stimuli in Study 3.

	Positive stimuli		Negative stimuli			
	*M*	*SD*	*M*	*SD*	*t*(79)	*p*
Valence	7.15	1.07	1.46	0.75	34.97	<0.001
Recognizability	6.82	0.44	3.77	1.33	21.85	<0.001
Familiarity	6.43	0.76	1.78	1.04	36.70	<0.001

### Results

#### Descriptive

Reaction times from error trials (4.5%) were excluded from the analysis. Two participants had error rates more than 2.5 SD above the mean. Excluding their data did not change the results, they were thus retained for analysis. Reaction times under 300 ms (0.04%) were replaced by 300 ms and reaction times above the threshold of 2.5 SD above the individual mean (2.8%) were replaced by that threshold (Wittenbrink, 2007). Reaction times indicated a significant priming effect: Responses in incongruent trials (*M* = 626.2 ms, *SD* = 59.5 ms) were slower than in congruent trials (*M* = 604.9 ms, *SD* = 58.9 ms), *t*(79) = 8.20, *p* < 0.001. Average indecisiveness was 2.74 (*SD* = 0.69). Hunger was not correlated with the priming effect, *r* = 0.01 *p* = 0.92, and was thus not included as a control variable.

#### Correlation Between Indecisiveness and Evaluative Priming Effect

We calculated an index for the individual priming effect by subtracting the average reaction time in congruent trials from that in incongruent trials per person. Higher values reflect a stronger priming effect. Indecisiveness was uncorrelated to the priming effect, τ = −0.02, *p* = 0.77, CI_95%_ [−0.26, 0.18]. The Bayes factor favored the null hypothesis, BF_01_ = 6.56.

### Additional Analyses

Additional analyses were conducted similar to the preceding studies (see ESM 3.1). The relationship between indecisiveness and the priming effect for each prime valence was calculated separately, as well as the priming effect for each participant’s personal favorite prime and per block. This was based on the reasoning that only stimuli of a certain valence, e.g., positive stimuli, might be affected by evaluation difficulty in indecisiveness (cf. van Randenborgh et al., 2010). Additionally, we repeated all analyses with the priming effect based on error rates instead of reaction times. Finally, we reran the correlation analysis between indecisiveness and the reaction time-based priming effect using the IS-Short and IS-AI. None of the additional analyses yielded a relationship in the expected direction.

### Discussion

In addition to evaluation difficulties based on explicit evaluations in Studies 1 through 2b, Study 3 tested whether indecisiveness was correlated with evaluations based on an implicit measure. Replicating the findings based on explicit ratings, a correlation between indecisiveness and evaluation difficulty was not supported. This adds further evidence to the observation that indecisiveness is independent of global evaluations of familiar everyday objects.

We used pictures of food as primes in order to ensure a strong evaluation (Lamote et al., 2004) as well as relevance for decision making as food is strongly connected to phylogenetically basic evaluation systems (see Chapman and Anderson, 2012). This makes the lack of an association between indecisiveness and the priming effect even more remarkable.

Evaluative priming entails certain disadvantages, especially low reliability (Bosson et al., 2000), which may impede the detection of correlations (Hofmann et al., 2005). Yet we chose this method in order to determine an implicit rating for each individual evaluation object. Other implicit measures (e.g., the IAT) assess evaluations of a whole target category (Greenwald et al., 1998) or suffer from similarly low reliability (Bosson et al., 2000). Another potential limitation of Study 3 is the low number of priming stimuli. This was a conscious decision to allow for collecting a sufficient number of reaction times per prime stimulus. We were thus able to analyze the favorite prime separately. Future studies could include larger numbers of prime stimuli to allow for generalization. Further, the negative priming stimuli showed lower recognizability. This calls into question the comparability of the different prime valances. However, a priming effect for the negative prime stimuli was present, which strongly indicates that the stimuli were recognizably negative and effective. Furthermore, the correlation with indecisiveness was not significant in the predicted direction for positive primes either. The difference in recognizability, therefore, did not seem to compromise internal validity.

## General Discussion

To know what one likes and dislikes, in other words, to evaluate objects, is an essential requirement for deciding (Heckhausen and Gollwitzer, 1987; Maio et al., 2013). Accordingly, definitions of indecisiveness generally include uncertainty about one’s own evaluations (Frost and Shows, 1993; Anderson, 2003).

Despite its relevance, this connection has not been empirically tested by separating evaluation from decision. Preceding studies have been inconsistent about the correlation between indecisiveness and the time needed to express a preference (e.g., Frost and Shows, 1993; Rassin et al., 2008), but these expressions of preference always required decisions. Additionally, the studies relied on small samples (*N* < 55) and used extreme group comparisons. Therefore, our studies are the first to directly look at evaluation behavior strictly separated from decisions. Across four studies, we did not find any systematic relation between indecisiveness and the indicators of evaluation difficulties focused on. This was true despite using different unobtrusive measurements of evaluation difficulties and different evaluation objects. The results held for evaluations based on explicit and implicit measures alike.

The model of indecisiveness proposed by Rassin (2007) poses difficulties in the evaluation of options as one cognitive mechanism that may contribute to indecisiveness. We defined these difficulties in terms of similarity and duration of evaluations and the evaluative priming effect reflecting the extremity, i.e., clarity of evaluations. However, our studies suggest that indecisiveness does not affect the ability to evaluate, at least when it comes to simple global evaluations of familiar everyday objects based on a salient dimension. Preceding research findings, which suggested a link between indecisiveness and evaluation difficulties seem to be inconsistent with the present results. However, these studies did not measure evaluation difficulty directly. For instance, van Randenborgh et al. (2009) observed increased indecisiveness after a rumination induction. Arguably, rumination entails more than a blocked access to subjective evaluations, e.g., negative mood (Watkins and Roberts, 2020). Interestingly, not only do our results contradict intuition, they are also in conflict with the self-perception of individuals high on indecisiveness. The Indecisiveness Scale item reflecting evaluation difficulties (“I always know exactly what I want,” reverse coded) is embraced as part of the indecisiveness construct just like the other items, lacking any psychometric hints on discrepancy judging by item-total-correlation (see ESM 4). In other words, a highly indecisive person will tend to state they often do not know what they want, while our results suggest that indecisiveness is unrelated to not knowing what one wants. This might be indicative of an underestimation of decision skills. In support of this idea, relationships between self-reported decision skills as captured by indecisiveness and more objective measures of decision process or outcome quality are weak at best. For example, Bavolar (2018) found only a very low correlation between Indecisiveness Scale scores and a more behavior-oriented measure of decision outcome quality. Similarly, Wood and Highhouse (2014) found a weak correlation between only one of five self-reported decision style dimensions and peer-reported decision quality. Other findings on decision-related processes as a function of indecisiveness have yielded inconsistent results. While some studies have found a relationship between indecisiveness and a preference for information about the chosen option (“information tunnel vision,” Rassin et al., 2008), others have failed to show such a relationship (Patalano and LeClair, 2011). Evidence regarding the association between decision-making quality and indecisiveness are thus inconclusive. One facet of indecisiveness may therefore be the underestimation of decision-making skills. The robust negative correlation between indecisiveness and self-esteem (Patalano and LeClair, 2011) is in line with this. However, while low self-esteem may plausibly lead to underestimating one’s decision skills in a right or wrong decision, it is less intuitive that it would affect decisions that are solely about liking or disliking something.

The presented studies include limitations that require consideration. The evaluation objects were familiar everyday objects (e.g., faces in Study 1) that were evaluated globally on a highly relevant dimension only (e.g., attractiveness in Study 1). Yet many decision-making contexts may contain a complex multi-dimensional rating object (e.g., Cacioppo and Berntson, 1994; Baron, 2008). This multi-dimensionality increases the trade-off difficulty (Dhar, 1997). It is possible that a multi-dimensional rating might produce the originally predicted correlation between indecisiveness and evaluation problems. In order to test this, future studies should vary the number of rating dimensions (see Ferrari and Dovidio, 2000), e.g., by including information about the price, prestige, healthiness, etc., of evaluation objects.

In a similar vein, the evaluation objects had no decision relevance (Studies 1 and 3) or only minor relevance, i.e., which chocolate bar to receive (Studies 2a and b). Possibly, indecisiveness may be related to evaluation difficulties only in high stake decisions. This is in line with the assumption by Rassin (2007) that decision importance moderates whether a decision causes indecisiveness. Future research should include evaluations in settings where the outcome is more relevant to participants. However, one hallmark of indecisiveness is that even trivial decisions are considered difficult (Frost and Shows, 1993).

In our studies based on explicit ratings we used rating times and rating standard deviations, two nonreactive measures of evaluation difficulties. They were derived from theoretical considerations (Dhar, 1997; Anderson, 2003; Schneider et al., 2015), yet had not been evaluated as indicators of evaluation difficulties. This is why both measures were included. The lack of a correlation between them suggests that they measure different constructs. Especially rating duration could conceivably reflect processes other than actual evaluation difficulties, e.g., avoidance: If evaluating objects gets harder with increasing indecisiveness, this uncomfortable task might intentionally be shortened (cf., Barkley-Levenson and Fox, 2016). However, this did not show consistently in our results either. Only Study 1 yielded a negative correlation between indecisiveness and rating time. The implicit measure assessing evaluation difficulty in Study 3 therefore provides a valuable complementary perspective. Despite the high relevance of implicit ratings for the decision-making processes (e.g., Galdi et al., 2012), this is, to the best of our knowledge, the first study to include implicit measures in the context of trait indecisiveness. Yet indecisiveness did not display the expected negative correlation with the strength of the implicit evaluation, replicating the findings based on explicit measures.

Despite the aforementioned limitations, the studies provide consistent evidence that indecisiveness is unrelated to difficulties with evaluations. The studies had sufficient power to find an effect (of *r* ≥ 0.31): Power was above 80% across studies and exceeded 90% in Studies 1 through 2b. Bayesian analyses were conducted to back up the null findings and the Bayes factors supported the results. There were no indications of a limited range in indecisiveness scores within our studies. A substantial proportion of each study’s sample had higher indecisiveness scores than the average score found in clinical samples known for elevated indecisiveness (e.g., obsessive-compulsive disorder or hoarding disorder, Steketee et al., 2003). Also, the means and standard deviations were in no way lower than those of non-clinical samples in earlier studies. The distribution showed a wide range of scores on both sides of the sample mean across all our studies. Details are given in ESM 5. The results are therefore applicable to a wide range of indecisiveness scores. However, a replication in a clinical sample known for more extreme indecisiveness scores would be a valuable addition.

If future studies should corroborate the findings of normal evaluations despite indecisiveness, implications for overcoming indecisiveness become apparent. Mindfulness interventions could focus on improving access to these subjective experiences. Mindfulness comprises a mental state characterized by focusing on the here and now and accepting whatever is experienced. It is thereby aimed to gain a better understanding of one’s own inner values after which to guide one’s actions (Kabat-Zinn and Hanh, 2009) and it is associated with less regret in decision making (Friese and Hofmann, 2016).

The question remains why intact evaluations do not translate into decisiveness. Our results underline that indecisiveness is not synonymous to not knowing what one likes or dislikes and that making satisfactory decisions requires more than that knowledge. Candidates for alternative explanations are lack of information and outcome uncertainty according to the model of indecisiveness (Rassin, 2007). In our paradigms, we chose familiar stimuli that provided all the information and entailed no outcome uncertainty to exclude these mechanisms as potential confounds. Possibly, in other real-life decisions these mechanisms may come to bear. Also post-decisional processes (Heckhausen and Gollwitzer, 1987) might provide an explanation. After a preference has been formed, the preferred alternative is enhanced by cognitive (Fujita et al., 2007) and motivational processes like cognitive dissonance reduction (Festinger, 1957), which is thought to facilitate effective choice and action (Harmon-Jones et al., 2015). Deviations in these processes could impede indecisive individuals’ decision making despite their initial evaluations being normal. These ideas should be explored in future studies.

## Conclusion

The current results provide initial evidence that indecisiveness is not based on evaluation difficulties, although decisions and evaluations are often treated interchangeably. Indecisiveness appears unrelated to difficulties evaluating familiar everyday objects globally, even when these evaluations determine what a person receives. Also at the implicit evaluation level, there seem to be no differences in evaluation difficulties contingent on indecisiveness, which – to our knowledge – has not been investigated before. This is particularly surprising because a negative self-perception of one’s evaluation abilities is part of the indecisiveness construct. As such, our results highlight the necessity to search for other cognitive mechanisms explaining indecisiveness and to scrutinize even highly face valid supposed reasons for it. Also, they have potential implications for overcoming indecisiveness by highlighting intact evaluations as a resource that may be made more accessible to benefit the decision-making process.

## Data Availability Statement

The raw data supporting the conclusions of this article will be made available by the authors, without undue reservation.

## Ethics Statement

Ethical review and approval was not required for the study on human participants in accordance with the local legislation and institutional requirements. The patients/participants provided their written informed consent to participate in this study.

## Author Contributions

HA designed the studies, which was advise by BE (Studies 1–3) and JB (Study 3). HA collected and analyzed the data, which was supervised by BE. HA created the first draft of the manuscript, which was revised by JB and BE. HA and JB agreed to the final version of the manuscript. All authors contributed to the article and approved the submitted version.

## Conflict of Interest

The authors declare that the research was conducted in the absence of any commercial or financial relationships that could be construed as a potential conflict of interest.

## Publisher’s Note

All claims expressed in this article are solely those of the authors and do not necessarily represent those of their affiliated organizations, or those of the publisher, the editors and the reviewers. Any product that may be evaluated in this article, or claim that may be made by its manufacturer, is not guaranteed or endorsed by the publisher.
