# Supplementary material for: “I Know What I Like” – Indecisiveness Is Unrelated to Behavioral Indicators of Evaluation Difficulties
Source: Front Psychol. 2021 Sep 16;12:710880. doi: 10.3389/fpsyg.2021.710880 (PMC8481952; doi:10.3389/fpsyg.2021.710880)
Supplement: Supplementary file 1 [file Data_Sheet_1.pdf]

Electronic Supplemental Materials

"I know what I like" - Indecisiveness is unrelated to behavioral indicators of evaluation difficulties

Electronic Supplemental Materials Study 1 (ESM 1)

1) Instructions and Materials for Study 1
1.1) Inquiry about sexual attraction

Do you feel more sexually attracted to men or to women? [1 = *clearly more to men* 2 = *slightly more to men* 3 = *slightly more to women* 4 = *clearly more to women*]

1.2) Portrait photo evaluation

Instructions:
You will now be presented with photos of faces. You are asked to rate these photos in terms of
- attractiveness
- pleasantness
- liking

Please try to make **quick, spontaneous** ratings based on your **personal impression**. There are no right or wrong answers.

Please answer the following questions according to your spontaneous personal impression and as quickly as possible.
Just drag the blue button to the point on the scale that best reflects your impression.

Items:
- How attractive do you personally think this face is?
- How pleasant do you personally think this face is?
- How much do you like this face?

[49 unit slider bar ranging from *not at all* to *extremely*]

Stimuli Portrait Photos (Corneille et al., 2005): Female and male portraits sample pictures

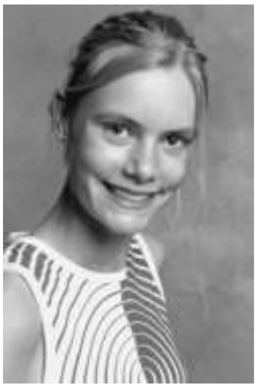
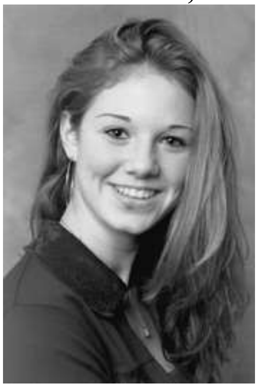
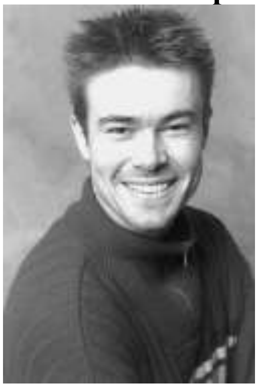
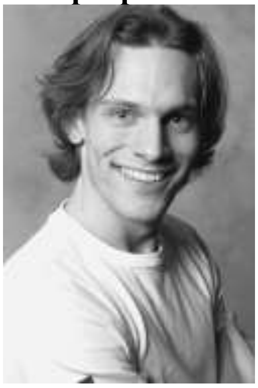

1.3) Indecisiveness Scale (Frost & Shows, 1993)

Instructions:
Please indicate the extent to which you agree to the following statements.

Items:

- I try to put off making decisions.
- I always know exactly what I want.
- I find it easy to make decisions.
- I have a hard time planning my free time.
- I like to be in a position to make decisions.
- Once I make a decision, I feel fairly confident that it is a good one.
- When ordering from a menu, I usually find it difficult to decide what to get.
- I usually make decisions quickly.
- Once I make a decision, I stop worrying about it.
- I become anxious when making a decision.
- I often worry about making the wrong decision.
- After I have chosen or decided something, I often believe I've made the wrong choice or decision.
- I do not get assignments done on time because I cannot decide what to do first.
- I have trouble completing assignments because I can't prioritize what is most important.
- It seems that deciding on the most trivial thing takes me a long time.

[1 = *strongly disagree* to 5 = *strongly agree*]

2) Additional analyses for Study 1

Because the results contradicted our predictions, we conducted additional analyses to test some of the boundary conditions which could conceivably have influenced the results. First of all we excluded participants who reported being distracted considerably (n = 2). We interpreted values higher than the midpoint of the scale as strong distraction. The correlations with indecisiveness did not change noticeably, $\tau_{\text{standard deviation}} = -.002$, $p = 1$, $\tau_{\text{rating time}} = -.18$, $p = .002$.
Another factor which could have influenced the assumed correlation is the congruence with the participants' sexual preference. It has been theorized that the assessment of attractiveness of the preferred sex is usually affected by sexual attraction, unlike the assessment of the non-preferred sex (DeBruine, 2004; Zebrowitz & Rhodes, 2002), which calls for a distinction between both types of ratings. On these grounds we repeated our analyses for the preferred and non-preferred sex separately. Further, as men and women differ in their reactions to images of the preferred sex (Cloutier et al., 2008) we also considered participants' sex. Therefore, we calculated a model in which indecisiveness (centered), participant sex (dummy coded) and their interaction predicted the standard deviation of the ratings for the preferred and non-preferred sex separately as predictors.
We repeated this analysis for the rating time. In order to reduce noise in ratings due to sexual orientation we excluded all participants who were not exclusively heterosexual. This applied to 15 women (9.9%) and six men (4.0%). All statistical values are displayed in Table 1. Even so, indecisiveness did not predict the standard deviation the ratings. However, the negative correlation with rating time also disappeared under these conditions. Only the participants' sex accounted for some of the variance within the standard deviation of the ratings. Women's ratings of the non-preferred sex had a larger standard deviation. But as there was no interaction with indecisiveness, this result is of no relevance to the question and will not be discussed further.
Due to an ongoing to debate about the most appropriate form of the Indecisiveness Scale (e.g., Lauderdale & Oakes, 2021), we repeated our main analyses using two important versions of the scale:

The 11-item version by Rassin et al. (2007, referred to as IS-Short), excluding all situation-specific items (e.g., “When ordering from a menu, I usually find it difficult to decide what to get”), and the Aversive Indecisiveness subscale identified by Lauderdales and Oakes (2021, referred to as IS-AI). The Positive Beliefs about Decision-Making subscale from the Lauderdales and Oakes (2021) study was not included because Aversive Indecsiveness is considered to be closer to the original definition of indecisiveness. In contrast, the Positive Beliefs about Decision-Making items appear to measure a different construct, correlating only modestly with Aversive Indecisiveness and uniquely predicting different constructs. Lauderdale and Oakes (2021) suggest they capture decisional self-efficacy, rather than indecisiveness. To avoid the inflation of Alpha error probability, we Bonferroni-corrected all *p*-values. Results are presented in Table 2. The correlation coefficients mirrored the main analyses results, except for the negative correlation between the 11-item version of the Indecisiveness Scale and rating no longer being significant after Bonferroni-correction.

Table 1

Regression of evaluation difficulty (rating standard deviation and rating time) on indecisiveness, sex, and their interaction in Study 1.

	Rating variability (SD)			Rating time (seconds)		
Preferred sex	β	*t*	*p*	β	*t*	*p*
Indec.	.21	1.85	.27	.05	0.42	1.00
Sex	.06	0.69	1.00	-.10	1.20	.92
Indec. x sex	.13	1.19	.95	-.09	0.81	1.00
Complete model	R^2	.03			.01	
	F	1.28			0.72	
Non-preferred sex	β	*t*	*p*	β	*t*	*p*
Indec.	-.16	1.52	.52	-.15	1.33	.76
Sex	.30	3.88**	< .001	-.01	0.12	1.00
Indec. x sex	.16	1.51	.52	.03	0.29	1.00
Complete model	R^2	.11			.02	
	F	5.88**			0.83	

Notes: Indec. = indecisiveness, sex = participant sex (0 = male, 1 = female); analyses should be interpreted cautiously due to deviation from normal distribution which could not be corrected by log-transformation; due to the exploratory nature of the analyses all *p*-values have been adjusted using the Bonferroni-correction for the number of models (4).

** $p < .01$

Table 2

Correlations between other Indecisiveness Scale versions and evaluation difficulty (rating standard deviation and rating time) in Study 1.

	1.	2.	3.	4.
1. IS Short	1			
2. IS AI	.73***	1		
3. Rating Time	-.13	-.17**	1	
4. Rating SD	-.02	.02	-.03	1

Notes: IS Short = Indecisiveness Scale, 11-item version (Rassin et al., 2007); IS AI = Indecisiveness Scale, Aversive Indecisiveness subscale (Lauderdales & Oakes, 2021); correlations based on Kendall's tau due to deviation from normal distribution; due to the exploratory nature of the analyses, all *p*-values have been adjusted using the Bonferroni-correction for the number analyses (4).

** $p < .01$ *** $p < .001$

Electronic Supplemental Materials Study 2a (ESM 2a)

1) Instructions and Materials for Study 2a
1.1) General instructions:

[Please note: original German instructions are included below each paragraph in italics]

In this study you are helping us to identify which chocolate varieties we should use as a reward in future studies. You will be asked to evaluate how much you would like to eat the chocolate varieties shown.

In dieser Studie helfen Sie uns abzuschätzen, welche Schokoladensorten wir in Zukunft zur Belohnung von Versuchsteilnehmer/innen verwenden sollen. Sie werden darum gleich gebeten zu bewerten, wie gern Sie die abgebildeten Schokoladensorten essen würden.

1.2) Presentation/Consequence manipulations

Sequential/with consequence condition:
Please rate each chocolate type one after the other. At the end you will receive one variety as a thank you. This corresponds to the variety that you rated the highest.
Please rate the chocolate in general, not according to your current appetite. If you don't feel like eating chocolate right now, please rate the chocolate as if you had an appetite for it. If you don't know a variety, just imagine how much you would like to eat it. It all depends on your spontaneous, personal evaluation, there are no right or wrong answers.
To do this, drag the slider with the mouse button pressed down to the position on the ray that best represents how much you would like to eat the illustrated varieties (see illustration).

Bitte bewerten Sie jede Schokoladensorte nacheinander. Am Ende bekommen Sie eine Sorte als Dankeschön. Diese ***entspricht der Sorte****, die Sie am* ***höchsten bewertet*** *haben.*
Bitte bewerten Sie die Schokolade im Allgemeinen, nicht nach Ihrem aktuellen Appetit. Wenn Sie gerade keinen Appetit auf Schokolade verspüren, bewerten Sie die Schokolade trotzdem so, als hätten Sie Appetit darauf. Sollten Sie eine Sorte nicht kennen, stellen Sie sich einfach vor, wie gern Sie diese essen würden. Es kommt dabei nur auf Ihre spontane, persönliche Bewertung an, es gibt keine richtigen oder falschen Antworten.
Dazu ziehen Sie den Schieberegler mit gedrückter Maustaste auf die Position auf dem Strahl, die am besten wiedergibt, wie gern Sie die abgebildeten Sorten essen würden (s. Abbildung).

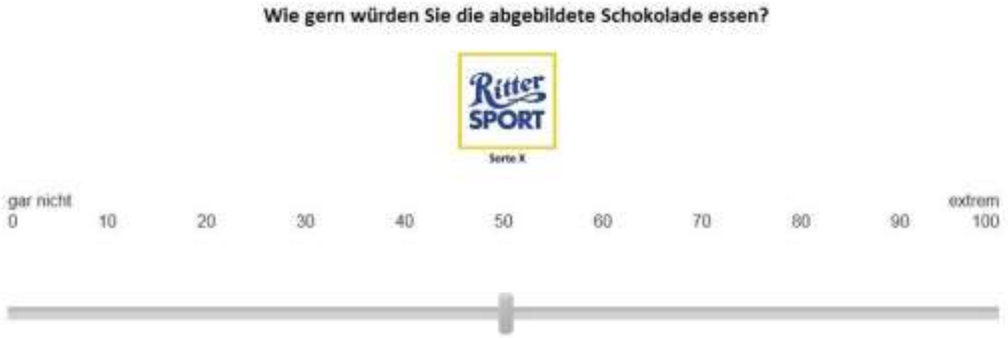

For example, if you would like to eat a variety very much, drag the slider to a high number.

Wenn Sie beispielsweise eine Sorte sehr gern essen würden, ziehen Sie den Schieberegler auf eine hohe Zahl.

Simultaneous/with consequence condition:
Please always evaluate two varieties in relation to each other. That means you indicate which variety you prefer. At the end you will receive one variety as a thank you. This corresponds to one of the varieties that you have rated higher.
Please rate the chocolate in general, not according to your current appetite. If you do not feel like chocolate at the moment, please rate the chocolate as if you had an appetite for it. If you don't know a variety, just imagine how much you would like to eat it. It all depends on your spontaneous, personal evaluation, there are no right or wrong answers.
To do this, drag the slider with the mouse button pressed down to the position on the beam that best reflects how much you prefer one variety over the other (see illustration).

Bitte bewerten Sie immer jeweils zwei Sorten im Verhältnis zueinander. D.h. Sie geben an, welche Sorte Sie bevorzugen. Am Ende bekommen Sie eine Sorte als Dankeschön. Diese entspricht ***einer der Sorten****, die Sie* ***höher bewertet*** *haben.*
Bitte bewerten Sie die Schokolade im Allgemeinen, nicht nach Ihrem aktuellen Appetit. Wenn Sie gerade keinen Appetit auf Schokolade verspüren, bewerten Sie die Schokolade trotzdem so, als hätten Sie Appetit darauf. Sollten Sie eine Sorte nicht kennen, stellen Sie sich einfach vor, wie gern Sie diese essen würden. Es kommt dabei nur auf Ihre spontane, persönliche Bewertung an, es gibt keine richtigen oder falschen Antworten.
Dazu ziehen Sie den Schieberegler mit gedrückter Maustaste auf die Position auf dem Strahl, die am besten wiedergibt, wie stark Sie eine Sorte vor der anderen bevorzugen (s. Abbildung).

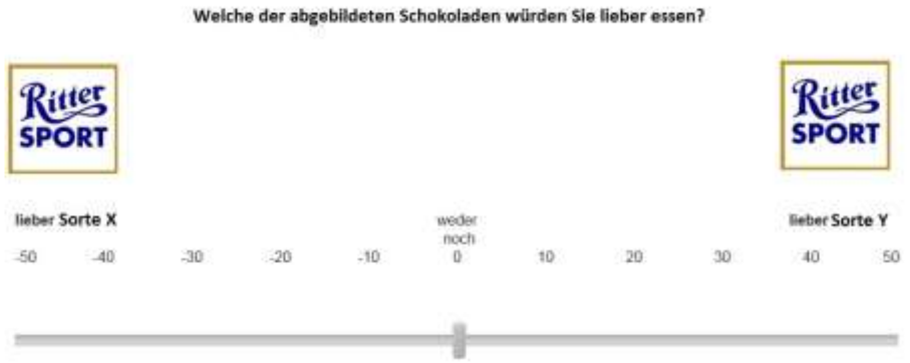

For example, if you prefer the left-hand variety very much, drag the slider further to the left side of the beam. If you would like to eat two varieties exactly the same, drag the slider to the middle of the beam.

Wenn Sie beispielsweise die linke Sorte sehr stark bevorzugen, ziehen Sie den Schieberegler weiter auf die linke Seite des Strahls. Wenn Sie zwei Sorten genau gleich gern essen würden, ziehen Sie den Schieberegler in die Mitte des Strahls.

Sequential/without consequence condition:
Please rate each chocolate type one after the other. Please note that this is a pre-test, so the varieties you rate are different from the ones you receive as a thank you at the end.
Please rate the chocolate in general, not according to your current appetite. If you do not feel an appetite for chocolate at the moment, please rate the chocolate as if you had an appetite for it. If you don't know a variety, just imagine how much you would like to eat it. It all depends on your spontaneous, personal evaluation, there are no right or wrong answers.
To do this, drag the slider with the mouse button pressed down to the position on the ray that best represents how much you would like to eat the illustrated varieties (see illustration).

Bitte bewerten Sie jede Schokoladensorte nacheinander. Bitte beachten Sie, dass dies ein Vortest ist, die von Ihnen bewerteten Sorten sind daher ***andere*** *als die, die Sie am Ende* ***als Dankeschön*** *erhalten.*
Bitte bewerten Sie die Schokolade im Allgemeinen, nicht nach Ihrem aktuellen Appetit. Wenn Sie gerade keinen Appetit auf Schokolade verspüren, bewerten Sie die Schokolade trotzdem so, als hätten Sie Appetit darauf. Sollten Sie eine Sorte nicht kennen, stellen Sie sich einfach vor, wie gern Sie diese essen würden. Es kommt dabei nur auf Ihre spontane, persönliche Bewertung an, es gibt keine richtigen oder falschen Antworten.
Dazu ziehen Sie den Schieberegler mit gedrückter Maustaste auf die Position auf dem Strahl, die am besten wiedergibt, wie gern Sie die abgebildeten Sorten essen würden (s. Abbildung).

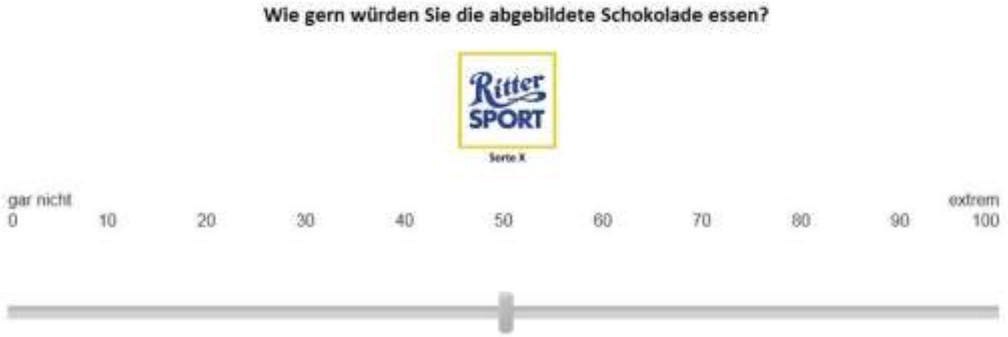

Wenn Sie beispielsweise eine Sorte sehr gern essen würden, ziehen Sie den Schieberegler auf eine hohe Zahl.

Wenn Sie beispielsweise eine Sorte sehr gern essen würden, ziehen Sie den Schieberegler auf eine hohe Zahl.

Simultaneous/without consequence condition:
Please always evaluate two varieties in relation to each other. That means you indicate which variety you prefer. Please note that this is a pre-test, the varieties you rate are therefore different from the ones you receive as a thank you at the end.
Please rate the chocolate in general, not according to your current appetite. If you do not feel an appetite for chocolate at the moment, please rate the chocolate as if you had an appetite for it. If you don't know a variety, just imagine how much you would like to eat it. It all depends on your spontaneous, personal evaluation, there are no right or wrong answers.
To do this, drag the slider with the mouse button pressed down to the position on the beam that best reflects how much you prefer one variety over the other (see illustration).

Bitte bewerten Sie immer jeweils zwei Sorten im Verhältnis zueinander. D.h. Sie geben an, welche Sorte Sie bevorzugen. Bitte beachten Sie, dass dies ein Vortest ist, die von Ihnen bewerteten Sorten sind daher ***andere*** *als die, die Sie am Ende* ***als Dankeschön*** *erhalten.*
Bitte bewerten Sie die Schokolade im Allgemeinen, nicht nach Ihrem aktuellen Appetit. Wenn Sie gerade keinen Appetit auf Schokolade verspüren, bewerten Sie die Schokolade trotzdem so, als hätten Sie Appetit darauf. Sollten Sie eine Sorte nicht kennen, stellen Sie sich einfach vor, wie gern Sie diese essen würden. Es kommt dabei nur auf Ihre spontane, persönliche Bewertung an, es gibt keine richtigen oder falschen Antworten.
Dazu ziehen Sie den Schieberegler mit gedrückter Maustaste auf die Position auf dem Strahl, die am besten wiedergibt, wie stark Sie eine Sorte vor der anderen bevorzugen (s. Abbildung).

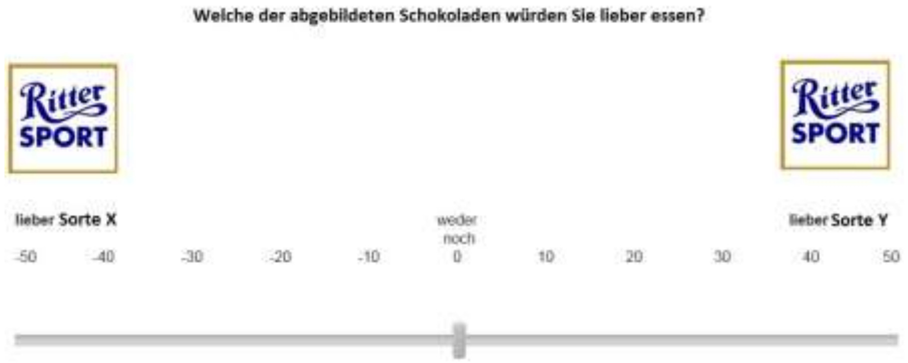

For example, if you prefer the left-hand variety very much, drag the slider further to the left side of the beam. If you would like to eat two varieties exactly the same, drag the slider to the middle of the beam.

Wenn Sie beispielsweise die linke Sorte sehr stark bevorzugen, ziehen Sie den Schieberegler weiter auf die linke Seite des Strahls. Wenn Sie zwei Sorten genau gleich gern essen würden, ziehen Sie den Schieberegler in die Mitte des Strahls.

1.3) Chocolate flavor evaluation

Instructions:
Please rate the chocolate in general, not according to your current appetite. If you do not feel an appetite for chocolate at the moment, please rate the chocolate as if you had an appetite for it. If you don't know a variety, just imagine how much you would like to eat it.
It all depends on your spontaneous, personal evaluation, there are no right or wrong answers.
To do this, drag the slider with the mouse button pressed down to the position on the beam that best reflects how much you prefer one variety over the other (see illustration).

Bitte bewerten Sie die Schokolade ***im Allgemeinen****, nicht nach Ihrem aktuellen Appetit. Wenn Sie gerade keinen Appetit auf Schokolade verspüren, bewerten Sie die Schokolade trotzdem so,* ***als hätten Sie Appetit darauf****. Sollten Sie eine Sorte* ***nicht kennen, stellen Sie sich einfach vor****, wie gern Sie diese essen würden.*
Es kommt dabei nur auf Ihre spontane, persönliche Bewertung an, es gibt keine richtigen oder falschen Antworten.
Dazu ziehen Sie den Schieberegler mit gedrückter Maustaste auf die Position auf dem Strahl, die am besten wiedergibt, wie stark Sie eine Sorte vor der anderen bevorzugen (s. Abbildung).

[100 unit slider bar, sequential presentation conditions: from 0 = *gar nicht* to 100 = *extrem*, simultaneous presentation conditions: from -50 = *lieber #name of flavor#* to 50 = *lieber #name of flavor#*]

Stimuli: Sample pictures of individual chocolate bars (pictures blurred for copyright reasons)

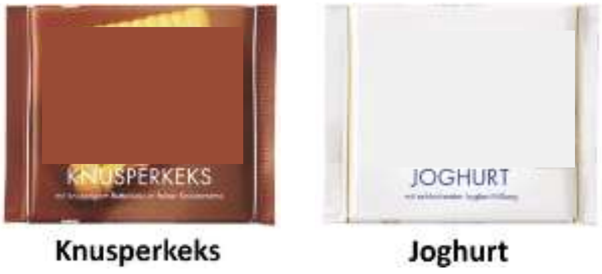

1.4) Choice

Instructions conditions with consequence:
Thank you very much for your reviews.
As a thank you, you will receive a bar of chocolate for your participation. Since it can be difficult to indicate preferences if not all options are known from the beginning, you now have the opportunity to make a choice from all varieties again. To select a variety, simply click on the corresponding image. When you have made your final decision, confirm your selection.
You can now freely choose a type of chocolate as a thank you.

Vielen Dank für Ihre Bewertungen.
Sie erhalten als Dankeschön eine Tafel Schokolade für Ihre Teilnahme. Da es schwierig sein kann, Präferenzen anzugeben, wenn nicht alle Optionen von Anfang an bekannt sind, haben Sie jetzt die Möglichkeit, erneut eine Wahl aus allen Sorten zu treffen. Um eine Sorte auszuwählen, klicken Sie einfach auf das entsprechende Bild. Wenn Sie sich endgültig entschieden haben, bestätigen Sie Ihre Auswahl.
Sie können nun eine Sorte Schokolade als Dankeschön frei auswählen.

Instructions conditions without consequence:
Thank you very much for your reviews.
As a thank you, you will receive a bar of chocolate for your participation. To select a variety, simply click on the corresponding picture. When you have made your final decision, confirm your selection.
You can now freely select a type of chocolate as a thank you.

Vielen Dank für Ihre Bewertungen.
Sie erhalten als Dankeschön eine Tafel Schokolade für Ihre Teilnahme. Um eine Sorte auszuwählen, klicken Sie einfach auf das entsprechende Bild. Wenn Sie sich endgültig entschieden haben, bestätigen Sie Ihre Auswahl.
Sie können nun eine Sorte Schokolade als Dankeschön frei auswählen.

Stimuli: Pictures of chocolate bar choice set
Conditions with consequence (pictures blurred for copyright reasons)

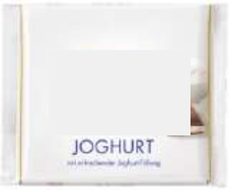

Joghurt

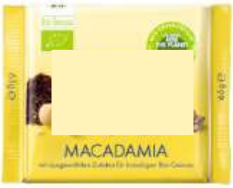

Bio Macadamia

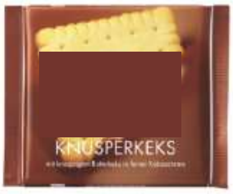

Knusperkeks

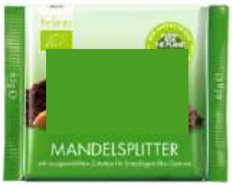

Bio Mandelsplitter

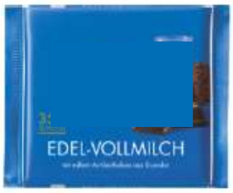

Edel-Vollmilch

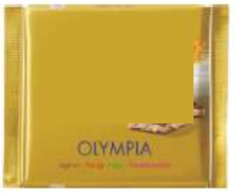

Olympia

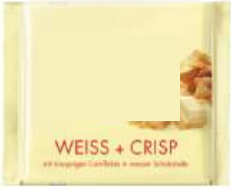

Weiß + Crisp

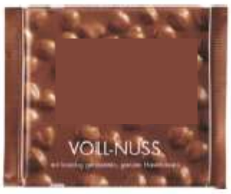

Voll-Nuss

Condition without consequence (pictures blurred for copyright reasons)

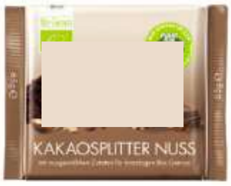

Bio Kakaosplitter-Nuss

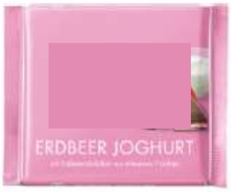

Erdbeer-Joghurt

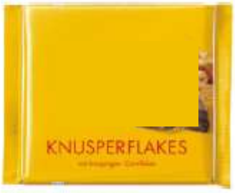

Knusperflakes

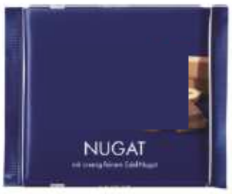

Nugat

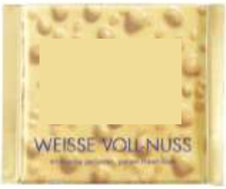

Weiße Voll-Nuss

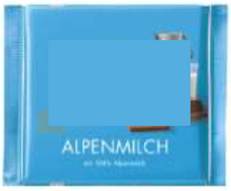

Alpenmilch

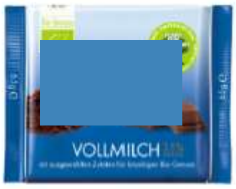

Bio Vollmilch

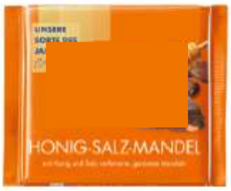

Honig-Salz-Mandel

INDECISIVENESS AND EVALUATION DIFFICULTIES

1.5) Indecisiveness Scale (Frost & Shows, 1993), German translation

Instructions:
Bitte machen Sie noch einige Angaben zu Ihrem ***allgemeinen*** Entscheidungsverhalten. Geben Sie an, inwieweit die folgenden Aussagen auf Sie zutreffen.

Items:
- Ich versuche Entscheidungen hinauszuschieben
- Ich weiß immer genau, was ich will
- Ich finde es einfach, Entscheidungen zu treffen
- Es fällt mir schwer, meine Freizeit zu planen
- Ich bin gerne in der Position Entscheidungen zu treffen
- Wenn ich einmal eine Entscheidung getroffen habe, bin ich ziemlich sicher, dass es eine gute Entscheidung war
- Wenn ich etwas von der Speisekarte bestelle, finde ich es meist schwierig zu entscheiden, was ich nehmen soll
- Ich treffe Entscheidungen meist schnell
- Wenn ich einmal eine Entscheidung getroffen habe, höre ich auf mir darüber Sorgen zu machen
- Ich werde ängstlich, wenn ich Entscheidungen treffen soll
- Ich mache mir oft Sorgen, dass ich die falsche Entscheidung treffen könnte
- Wenn ich etwas ausgewählt oder entschieden habe, habe ich oft das Gefühl die falsche Wahl oder Entscheidung getroffen zu haben
- Ich bekomme Aufgaben nicht pünktlich bearbeitet, weil ich mich nicht entscheiden kann, was ich zuerst tun soll
- Ich habe Probleme Aufgaben fertigzustellen, weil ich nicht die Priorität setzen kann, was am wichtigsten ist
- Scheinbar brauche ich lange, um selbst die trivialsten Entscheidungen zu treffen

[1 = *starke Ablehnung* to 5 = *starke Zustimmung*]

1.6) Choice difficulty

Instructions:
Please answer a few more questions about the decision you have just made:

Bitte beantworten Sie zunächst noch einige Fragen zu Ihrer gerade getroffenen Entscheidung:

Items:
- How difficult was it for you to decide which chocolate to choose?
- How sure were you about the chocolate you chose?
- How much would you have liked to avoid the choice of chocolate?
- How much would you have liked the opportunity to change your mind again?
- How satisfied are you with your choice of chocolate?
- How much do you regret not having chosen another chocolate?

[0 = *not at all to* 6 = *very much*]

- Wie schwer ist Ihnen die Entscheidung für die gewählte Schokolade gefallen?
- Wie sicher waren Sie sich bei der Entscheidung für die gewählte Schokolade?
- Wie gern hätten Sie die Entscheidung für die gewählte Schokolade vermieden?
- Wie gern hätten Sie noch einmal die Gelegenheit, sich für eine andere Schokolade

umzuentscheiden?

- Wie zufrieden sind Sie mit Ihrer Entscheidung für die gewählte Schokolade?
- Wie stark bedauern Sie, sich nicht für eine andere Schokolade entschieden zu haben?

[0 = *gar nicht* to 6 = *sehr*]

2) Additional analyses for Study 2a

We repeated the analyses while adjusting the level of significance using a Bonferroni-correction, i.e. dividing the significance level by the number of additional tests run on the same hypothesis (2). We first repeated the analyses without participants who had failed the attention check (n = 72). Doing this did not change the pattern of the results for either indicator of evaluation difficulty (standard deviation of the ratings and rating time). We could still not find any significant correlations between evaluation difficulty and indecisiveness or any of the interaction terms of indecisiveness, all $|\beta| < .13$, ns.
Further, we repeated the analyses without participants who were unable to recall correctly whether their rating had consequence or not (n = 69), which might undermine the validity of the manipulation. When applying this criterion there was still no significant correlation between evaluation difficulty and indecisiveness or any of its interaction terms, all $|\beta| < .28$, ns.
As in Study 1, we repeated our main analyses using the IS-Short and the IS-AI. To avoid the inflation of Alpha error probability, we Bonferroni-corrected all p-values. Results are presented in Table 3. As in the main analysis, only presentation significantly predicted rating standard deviation and rating time.

Table 3

Regression of evaluation difficulty (rating standard deviation and rating time) on indecisiveness, consequence of ratings, presentation, and their interactions in Study 2a, using other Indecisiveness Scale versions.

	Rating variability (SD)			Rating time (seconds)		
IS Short	β	*t*	*p*	β	*t*	*p*
Indec.	.02	0.17	1.00	.24	2.18	.12
Cons.	.00	0.03	1.00	-.06	-0.71	1.00
Pres.	-.30	-3.11	.008	.39	4.40	< .001
Indec. x Cons.	-.07	-0.53	1.00	-.21	-1.66	.40
Indec. x Pres.	-.02	-0.12	1.00	-.04	-0.36	1.00
Cons. x Pres.	-.05	-0.38	1.00	.15	1.31	.77
Indec. x Cons. x Pres.	.03	0.19	1.00	.11	0.81	1.00
Complete model	R^2	.12			.26	
	F	3.67**			9.54***	
IS AI	β	*t*	*p*	β	*t*	*p*
Indec.	-.06	-0.53	1.00	.24	2.21	.11
Cons.	.02	0.25	1.00	-.09	-0.95	1.00
Pres.	-.30	-3.05	.012	.38	4.33	< .001
Indec. x Cons.	.09	0.63	1.00	-.28	-2.28	.10
Indec. x Pres.	-.02	-0.18	1.00	-.06	-0.52	1.00
Cons. x Pres.	-.07	-0.57	1.00	.16	1.48	.56
Indec. x Cons. x Pres.	-.07	-0.48	1.00	.16	1.21	.90
Complete model	R^2	.12			.26	
	F	3.83**			9.54***	

Notes: IS Short = Indecisiveness Scale, 11-item version (Rassin et al., 2007); IS AI = Indecisiveness Scale, Aversive Indecisiveness subscale (Lauderdales & Oakes, 2021); Indec. = indecisiveness Cons. = consequence of the ratings (0 = without consequence, 1 = with consequence); Pres. = presentation of options (0 = simultaneously, 1 = sequentially; due to the exploratory nature of the analyses all *p*-values have been adjusted using the Bonferroni-correction for the number of models (4).

** $p < .01$ *** $p < .001$

Electronic Supplemental Materials Study 2b (ESM 2b)

1) Instructions and Materials for Study 2b
1.1) General instructions

[Please note: original German instructions are included below each paragraph in italics]

In this study you are helping us to identify which chocolate varieties we should use as a reward in future studies. You will be asked to evaluate how much you would like to eat the chocolate varieties shown.

In dieser Studie helfen Sie uns abzuschätzen, welche Schokoladensorten wir in Zukunft zur Belohnung von Versuchsteilnehmer/innen verwenden sollen. Sie werden darum gleich gebeten zu bewerten, wie gern Sie die abgebildeten Schokoladensorten essen würden.

1.2) Presentation/Consequence manipulations

Sequential/with consequence condition:
Please rate each chocolate type one after the other. At the end you will receive one variety as a thank you. This corresponds to the variety that you rated the highest.
Please rate the chocolate in general, not according to your current appetite. If you don't feel like eating chocolate right now, please rate the chocolate as if you had an appetite for it.
It all depends on your spontaneous, personal evaluation, there are no right or wrong answers.
Please drag the picture of the chocolate to the place that corresponds to your rating. If you would like to eat the variety, drag the picture to the right side (see fig. 1), if you do not like the variety, drag it to the left side (see fig. 2).

Bitte bewerten Sie jede Schokoladensorte nacheinander. Am Ende bekommen Sie eine Sorte als Dankeschön. Diese entspricht der Sorte, die Sie am höchsten bewertet haben.
Bitte bewerten Sie die Schokolade ***im Allgemeinen****, nicht nach Ihrem aktuellen Appetit. Wenn Sie gerade keinen Appetit auf Schokolade verspüren, bewerten Sie die Schokolade trotzdem so,* ***als hätten Sie Appetit darauf****.*
Es kommt dabei nur auf Ihre spontane, persönliche Bewertung an, es gibt keine richtigen oder falschen Antworten.
Bitte ziehen Sie das Bild von der Schokolade auf die Stelle, die Ihrer Bewertung entspricht. Möchten Sie die Sorte gern essen, ziehen Sie das Bild also auf die rechte Seite (s. Abb. 1), möchten Sie die Sorte nicht gern essen, ziehen Sie es auf die linke Seite (s. Abb. 2).

[redacted]

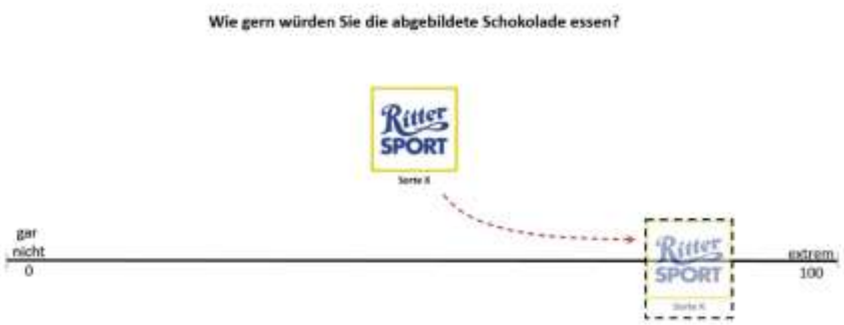

[redacted] Figure 1 [*Abbildung 1*].

[redacted]

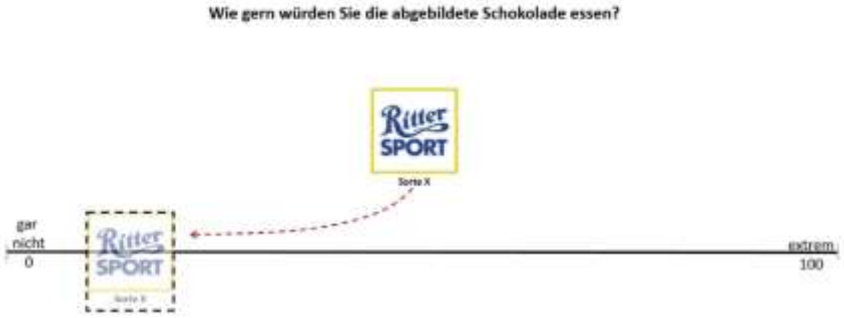

[redacted] Figure 2 [*Abbildung 2*].

It does not matter whether you drag the image exactly on the axis, above or below it. Only the horizontal position is important, i.e. how far to the right or left the picture of the chocolate is placed at the end. The axis is only for orientation.
Please make yourself familiar with the handling of the scale on the next page and click on Next to get to the actual ratings.

Es ist dabei egal, ob Sie das Bild genau auf die Achse, darüber oder darunter ziehen. Wichtig ist nur die horizontale Position, also wie weit rechts oder links das Bild von der Schokolade am Ende platziert ist. Die Achse dient nur zur Orientierung.
Bitte machen Sie sich auf der nächsten Seite mit der Handhabung der Skala vertraut und klicken Sie auf Weiter um zu den eigentlichen Bewertungen zu gelangen.

Simultaneous/with consequence condition:
Please always evaluate two varieties in relation to each other. That means you should rate the preferred variety higher. At the end you will get one variety as a thank you. This corresponds to the variety that you have rated the highest.
Please rate the chocolate in general, not according to your current appetite. If you don't feel like chocolate right now, please rate the chocolate as if you had an appetite for it.
It all depends on your spontaneous, personal evaluation, there are no right or wrong answers.
Please drag the picture of the chocolate to the place that corresponds to your rating. If you would like to eat the variety, drag the picture to the right side (see fig. 1), if you do not like the variety, drag it to

the left side (see fig. 2).

Bitte bewerten Sie immer jeweils zwei Sorten im Verhältnis zueinander. D.h. die bevorzugte Sorte sollten Sie höher bewerten. Am Ende bekommen Sie eine Sorte als Dankeschön. Diese entspricht der Sorte, die Sie am höchsten bewertet haben.
Bitte bewerten Sie die Schokolade ***im Allgemeinen****, nicht nach Ihrem aktuellen Appetit. Wenn Sie gerade keinen Appetit auf Schokolade verspüren, bewerten Sie die Schokolade trotzdem so,* ***als hätten Sie Appetit darauf****.*
Es kommt dabei nur auf Ihre spontane, persönliche Bewertung an, es gibt keine richtigen oder falschen Antworten.
Bitte ziehen Sie das Bild von der Schokolade auf die Stelle, die Ihrer Bewertung entspricht. Möchten Sie die Sorte gern essen, ziehen Sie das Bild also auf die rechte Seite (s. Abb. 1), möchten Sie die Sorte nicht gern essen, ziehen Sie es auf die linke Seite (s. Abb. 2).

[redacted]

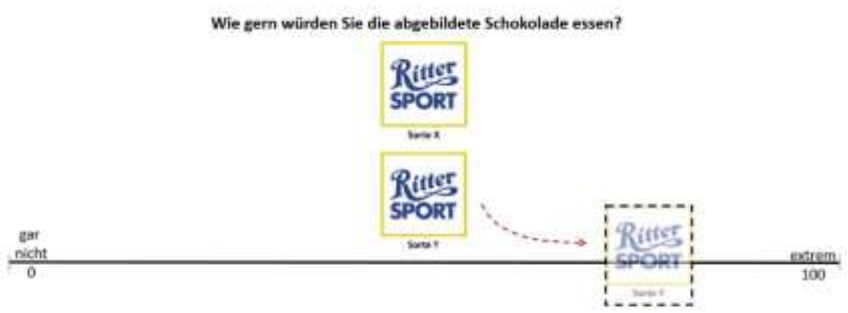

[redacted] Figure 1 [*Abbildung 1*].

[redacted]

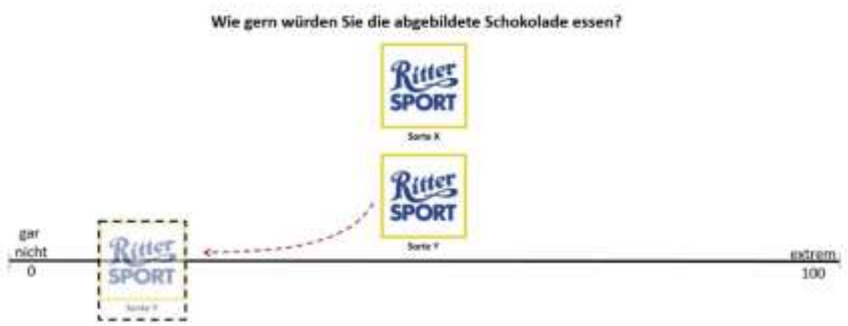

[redacted] Figure 2 [*Abbildung 2*].

It does not matter whether you drag the image exactly on the axis, above or below it. Only the horizontal position is important, i.e. how far to the right or left the picture of the chocolate is placed at the end. The axis is only for orientation.
Please always rate both varieties in this way. Your rating will also reflect which one you prefer: You should always place the preferred one further to the right than the non-preferred one. In the first example (see Fig. 3) the preferred grade is X, in the second (see Fig. 4) the preferred grade is Y. The images can also overlap (see fig. 3) or you can place one image above and one below the axis (see

fig. 4). If you don't prefer any type, both images should be placed at the same height on the axis.

Es ist dabei egal, ob Sie das Bild genau auf die Achse, darüber oder darunter ziehen. Wichtig ist nur die horizontale Position, also wie weit rechts oder links das Bild von der Schokolade am Ende platziert ist. Die Achse dient nur zur Orientierung.
Bitte bewerten Sie auf diese Weise immer beide Sorten. Ihre Bewertung spiegelt somit gleichzeitig wider, welche Sorte Sie bevorzugen: Die bevorzugte Sorte sollen Sie also immer weiter rechts als die nicht bevorzugte platzieren. Im ersten Beispiel (s. Abb. 3) ist die bevorzugte Sorte X, im zweiten (s. Abb. 4) ist die bevorzugte Sorte Y. Die Bilder können sich dabei auch überlappen (s. Abb. 3) oder Sie können ein Bild über und ein Bild unter der Achse platzieren (s. Abb. 4). Wenn Sie keine Sorte bevorzugen, sollten beide Bilder auf der gleichen Höhe der Achse platziert werden.

[redacted]

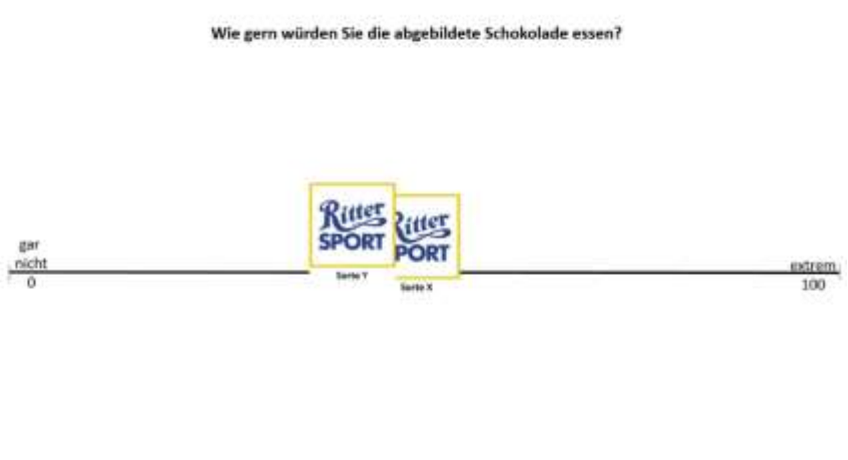

[redacted] Figure 3 [*Abbildung 3*].

[redacted]

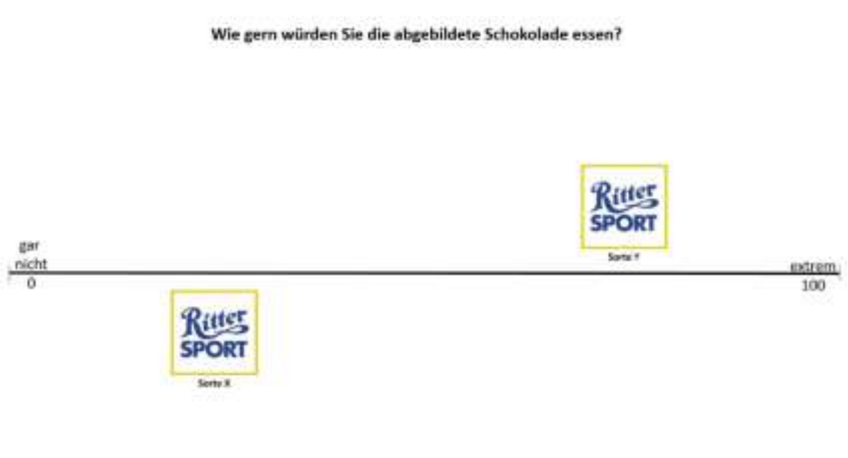

[redacted] Figure 4 [*Abbildung 4*].

Please make yourself familiar with the handling of the scale on the next page and click on Next to get to the actual ratings.

Bitte machen Sie sich auf der nächsten Seite mit der Handhabung der Skala vertraut und klicken Sie auf Weiter um zu den eigentlichen Bewertungen zu gelangen.

Sequential/without consequence condition:
Please rate each chocolate type one after the other. Please note that this is a pre-test, so the varieties

you rate are different from the ones you receive as a thank you at the end.
Please rate the chocolate in general, not according to your current appetite. If you do not feel an appetite for chocolate at the moment, please rate the chocolate as if you had an appetite for it.
It all depends on your spontaneous, personal evaluation, there are no right or wrong answers.
Please drag the picture of the chocolate to the place that corresponds to your rating. If you would like to eat the variety, drag the picture to the right side (see fig. 1), if you do not like the variety, drag it to the left side (see fig. 2).

Bitte bewerten Sie jede Schokoladensorte nacheinander. Bitte beachten Sie, dass dies ein Vortest ist, die von Ihnen bewerteten Sorten sind daher andere als die, die Sie am Ende als Dankeschön erhalten. Bitte bewerten Sie die Schokolade ***im Allgemeinen****, nicht nach Ihrem aktuellen Appetit. Wenn Sie gerade keinen Appetit auf Schokolade verspüren, bewerten Sie die Schokolade trotzdem so,* ***als hätten Sie Appetit darauf****.*
Es kommt dabei nur auf Ihre spontane, persönliche Bewertung an, es gibt keine richtigen oder falschen Antworten.
Bitte ziehen Sie das Bild von der Schokolade auf die Stelle, die Ihrer Bewertung entspricht. Möchten Sie die Sorte gern essen, ziehen Sie das Bild also auf die rechte Seite (s. Abb. 1), möchten Sie die Sorte nicht gern essen, ziehen Sie es auf die linke Seite (s. Abb. 2).

[redacted]

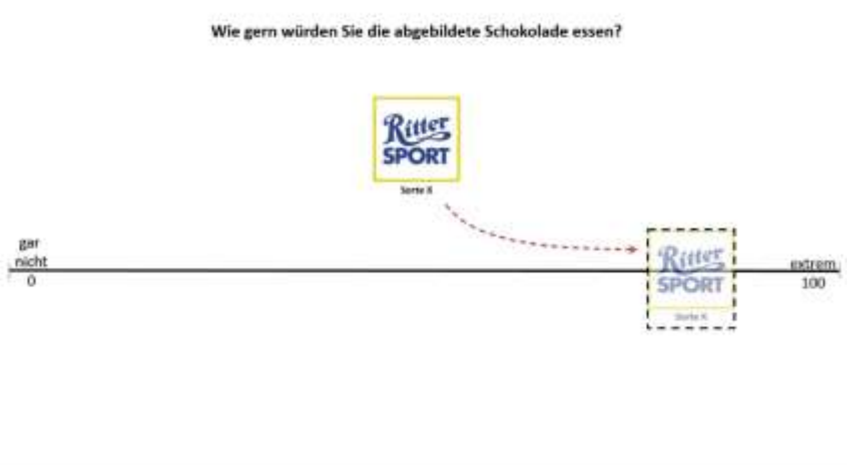

[redacted] Figure 1 [*Abbildung 1*].

[redacted]

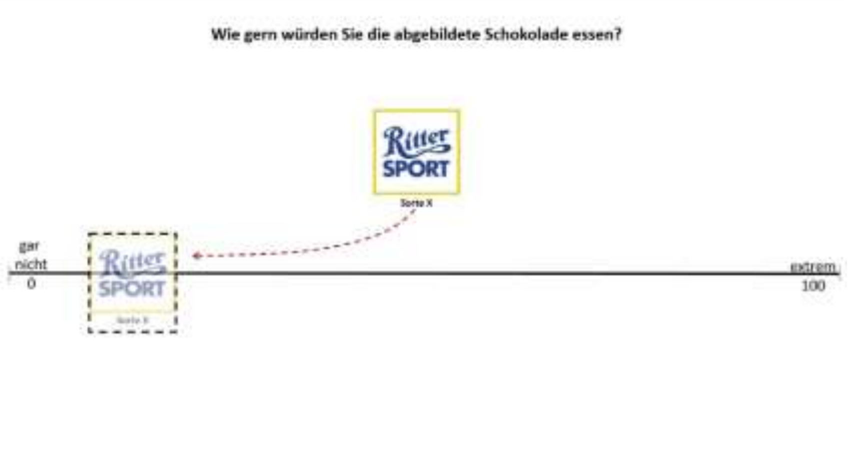

[redacted] Figure 2 [*Abbildung 2*].

It does not matter whether you drag the image exactly on the axis, above or below it. Only the horizontal position is important, i.e. how far to the right or left the picture of the chocolate is placed at the end. The axis is only for orientation.

Please make yourself familiar with the handling of the scale on the next page and click on Next to get to the actual ratings.

Es ist dabei egal, ob Sie das Bild genau auf die Achse, darüber oder darunter ziehen. Wichtig ist nur die horizontale Position, also wie weit rechts oder links das Bild von der Schokolade am Ende platziert ist. Die Achse dient nur zur Orientierung.
Bitte machen Sie sich auf der nächsten Seite mit der Handhabung der Skala vertraut und klicken Sie auf Weiter um zu den eigentlichen Bewertungen zu gelangen.

Simultaneous/without consequence condition:
Please always evaluate two varieties in relation to each other. That means you should rate the preferred variety higher. Please note that this is a pre-test, the varieties you rate are therefore different from the ones you receive as a thank you at the end.
Please rate the chocolate in general, not according to your current appetite. If you do not feel an appetite for chocolate at the moment, please rate the chocolate as if you had an appetite for it.
It all depends on your spontaneous, personal evaluation, there are no right or wrong answers.
Please drag the picture of the chocolate to the position that corresponds to your rating. If you would like to eat the variety, drag the picture to the right side (see fig. 1), if you do not like the variety, drag it to the left side (see fig. 2).

Bitte bewerten Sie immer jeweils zwei Sorten im Verhältnis zueinander. D.h. die bevorzugte Sorte sollten Sie höher bewerten. Bitte beachten Sie, dass dies ein Vortest ist, die von Ihnen bewerteten Sorten sind daher andere als die, die Sie am Ende als Dankeschön erhalten.
Bitte bewerten Sie die Schokolade im Allgemeinen, nicht nach Ihrem aktuellen Appetit. Wenn Sie gerade keinen Appetit auf Schokolade verspüren, bewerten Sie die Schokolade trotzdem so, als hätten Sie Appetit darauf.
Es kommt dabei nur auf Ihre spontane, persönliche Bewertung an, es gibt keine richtigen oder falschen Antworten.
Bitte ziehen Sie das Bild von der Schokolade auf die Stelle, die Ihrer Bewertung entspricht. Möchten Sie die Sorte gern essen, ziehen Sie das Bild also auf die rechte Seite (s. Abb. 1), möchten Sie die Sorte nicht gern essen, ziehen Sie es auf die linke Seite (s. Abb. 2).

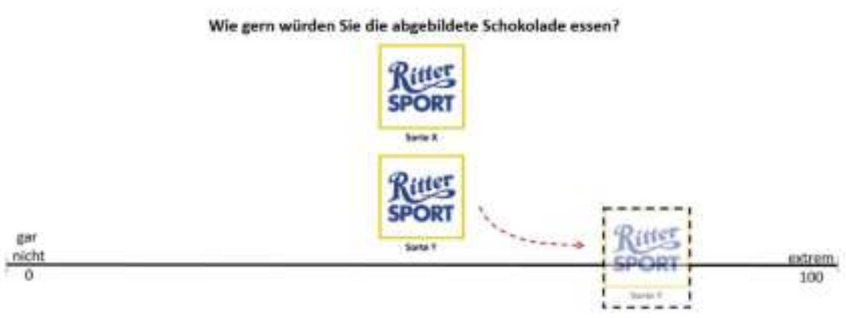

Figure 1 [*Abbildung 1*].

[redacted]

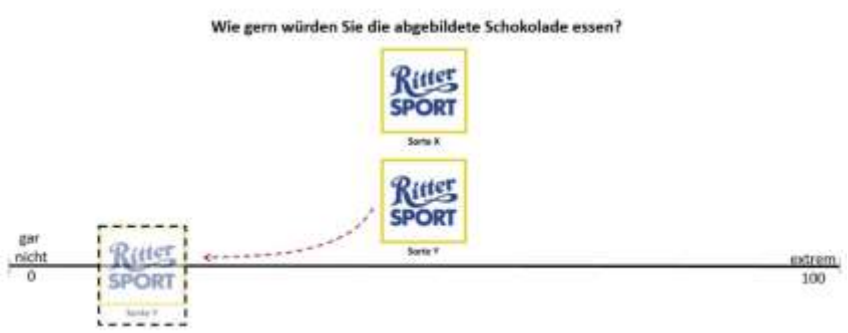

[redacted] Figure 2 [*Abbildung 2*].

It does not matter whether you drag the image exactly on the axis, above or below it. Only the horizontal position is important, i.e. how far to the right or left the picture of the chocolate is placed at the end. The axis is only for orientation.
Please always rate both varieties in this way. Your rating will also reflect which one you prefer: You should always place the preferred one further to the right than the non-preferred one. In the first example (see Fig. 3) the preferred grade is X, in the second (see Fig. 4) the preferred grade is Y. The images can also overlap (see fig. 3) or you can place one image above and one below the axis (see fig. 4). If you don't prefer any type, both images should be placed at the same height on the axis.

Es ist dabei egal, ob Sie das Bild genau auf die Achse, darüber oder darunter ziehen. Wichtig ist nur die horizontale Position, also wie weit rechts oder links das Bild von der Schokolade am Ende platziert ist. Die Achse dient nur zur Orientierung.
Bitte bewerten Sie auf diese Weise immer beide Sorten. Ihre Bewertung spiegelt somit gleichzeitig wider, welche Sorte Sie bevorzugen: Die bevorzugte Sorte sollen Sie also immer weiter rechts als die nicht bevorzugte platzieren. Im ersten Beispiel (s. Abb. 3) ist die bevorzugte Sorte X, im zweiten (s. Abb. 4) ist die bevorzugte Sorte Y. Die Bilder können sich dabei auch überlappen (s. Abb. 3) oder Sie können ein Bild über und ein Bild unter der Achse platzieren (s. Abb. 4). Wenn Sie keine Sorte bevorzugen, sollten beide Bilder auf der gleichen Höhe der Achse platziert werden.

[redacted]

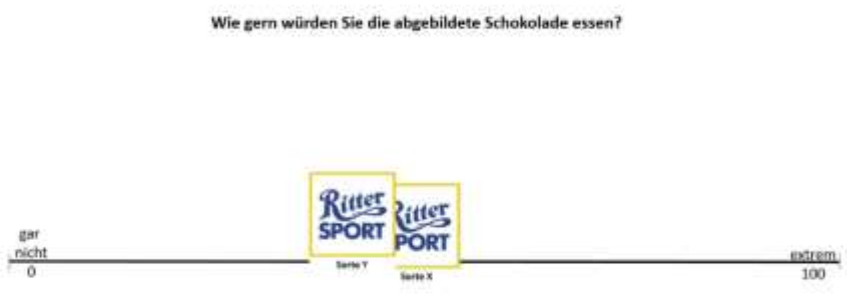

[redacted] Figure 3 [*Abbildung 3*].

[redacted]

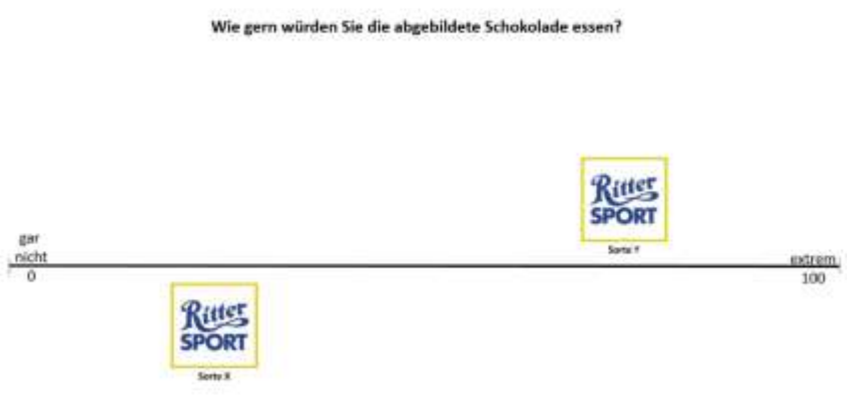

[redacted] Figure 4 [*Abbildung 4*].

Please make yourself familiar with the handling of the scale on the next page and click on Next to get to the actual ratings.

Bitte machen Sie sich auf der nächsten Seite mit der Handhabung der Skala vertraut und klicken Sie auf Weiter um zu den eigentlichen Bewertungen zu gelangen.

1.3) Chocolate flavor evaluation

Instructions:
Cf. Study 2a

[100 unit drag and drop bar from 0 = *gar nicht* to 100 = *extrem*, simultaneous presentation]

Stimuli: Pictures of individual chocolate bars
Cf. Study 2a

1.4) Indecisiveness Scale (Frost & Shows, 1993), German translation

Cf. Study 2a

1.5) Choice difficulty

Cf. Study 2a

2) Additional analyses for Study 2b

We conducted the same additional analyses as in Study 2a in order to scrutinize the negative results concerning the correlations with indecisiveness. After excluding participants who had not passed the attention check (n = 52), we repeated the analyses looking at a relationship between evaluation difficulty (standard deviation of the ratings and rating time) and indecisiveness or an interaction term of indecisiveness. No relationships were found, all $|\beta| < .21$, *ns*.
Further, we repeated the same analyses excluding participants who had failed the manipulation check (n = 62), as the validity of the manipulation was threatened for these participants. Even under these conditions no significant relationships between the indicators of evaluation difficulty and indecisiveness or any interaction terms of indecisiveness could be found, all $|\beta| < .23$, *ns*.
As in the preceding studies, main analyses were repeated with the IS-Short and the IS-AI. Again, we

Bonferroni-corrected all *p*-values. Results are depicted in Table 4. As reported in the main analysis, no predictor was significantly associated with either indicator of evaluation difficulty.

Table 4

Regression of evaluation difficulty (rating standard deviation and rating time) on indecisiveness, consequence of ratings, presentation, and their interactions in Study 2b, using other Indecisiveness Scale versions.

	Rating variability (SD)			Rating time (seconds)		
IS Short	β	*t*	*p*	β	*t*	*p*
Indec.	-.09	-0.59	1.00	-.02	-0.14	1.00
Cons.	.02	0.23	1.00	-.10	-0.98	1.00
Pres.	.09	0.95	1.00	-.04	-0.40	1.00
Indec. x Cons.	.02	0.17	1.00	.06	0.47	1.00
Indec. x Pres.	.08	0.55	1.00	.18	1.33	.74
Cons. x Pres.	-.12	-0.99	1.00	.13	1.07	1.00
Indec. x Cons. x Pres.	-.03	-0.21	1.00	-.08	-0.60	1.00
Complete model	R^2	.01			.03	
	F	0.29			0.97	
IS AI	β	*t*	*p*	β	*t*	*p*
Indec.	.06	0.42	1.00	-.12	-0.83	1.00
Cons.	.02	0.24	1.00	-.10	-0.99	1.00
Pres.	.10	0.98	1.00	-.03	-0.31	1.00
Indec. x Cons.	-.02	-0.14	1.00	.05	0.33	1.00
Indec. x Pres.	.03	0.22	1.00	.25	1.70	.36
Cons. x Pres.	-.13	-1.07	1.00	.13	1.10	1.00
Indec. x Cons. x Pres.	.00	0.00	1.00	-.13	-0.89	1.00
Complete model	R^2	.01			.03	
	F	0.39			0.78	

Notes: IS Short = Indecisiveness Scale, 11-item version (Rassin et al., 2007); IS AI = Indecisiveness Scale, Aversive Indecisiveness subscale (Lauderdales & Oakes, 2021); Indec. = indecisiveness Cons. = consequence of the ratings (0 = without consequence, 1 = with consequence); Pres. = presentation of options (0 = simultaneously, 1 = sequentially; due to the exploratory nature of the analyses all *p*-values have been adjusted using the Bonferroni-correction for the number of models (4).

Electronic Supplemental Materials Study 3 (ESM 3)

1) Instructions and Materials for Study 3
1.1) Evaluative priming task

[Please note: original German instructions are included below each paragraph in italics]

Instructions beginning:
Many thanks for your participation
Your task is to classify a word as positive or negative as quickly as possible. First, a cross will appear in the middle of the screen where the word appears. Then a picture will appear briefly at the same place and then the word to be judged will follow. Pay attention to both the picture and the word, but react only to the word. To do this, press the left mouse button for positive words and the right mouse button for negative words. Please leave the mouse on the rectangular base. React as quickly and accurately as possible. You will first go through a number of exercises which will not be evaluated. Please place your left index finger on the left mouse button and your right index finger on the right mouse button. Then press the right mouse button to get to the practice runs.

Vielen Dank für Ihre Teilnahme
Ihre Aufgabe besteht darin, so schnell wie möglich ein Wort als positiv oder negativ einzustufen. Zunächst wird in der Mitte des Bildschirms ein Kreuz erscheinen, wo das Wort auftaucht. Dann wird an der gleichen Stelle kurz ein Bild eingeblendet und darauf folgt das zu beurteilende Wort. Achten Sie sowohl auf das Bild, als auch auf das Wort, aber reagieren Sie ausschließlich auf das Wort. Dazu drücken Sie bei positive Wörtern die linke Maustaste, bei negative Wörtern auf die rechte Maustaste. Bitte lassen Sie die Maus auf der rechteckigen Unterlage. Reagieren Sie so schnell und akkurat wie möglich. Sie durchlaufen zunächst einige Übungsdurchgänge, die nicht gewertet warden. Platzieren Sie bitte Ihren linken Zeigefinger auf der linken Maustaste und Ihren rechten Zeigefinger auf der rechten Maustaste. Drücken Sie dann die rechte Maustaste um zu den Übungsdurchgängen zu gelangen.

Instructions after practice trials:
You have now completed the exercise. If you have any questions, please contact the test management quietly. Otherwise, please place your left index finger on the left mouse button and your right index finger on the right mouse button. Then press the right mouse button to start.

Sie haben nun den Übungsdurchgang beendet. Falls Sie Fragen haben, wenden Sie sich bitte leise an die Versuchsleitung. Ansonsten platzieren Sie bitte Ihren linken Zeigefinger auf der linken Maustaste und Ihren rechten Zeigefinger auf der rechten Maustaste. Drücken Sie dann die rechte Maustaste um zu beginnen.

Visual primes: Food pictures

Positive Primes (Blechert et al., 2014)

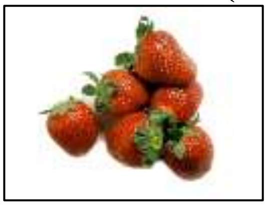
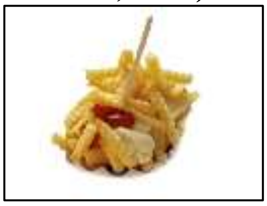
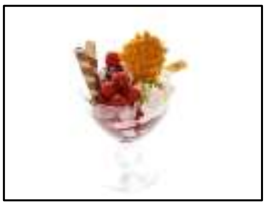

Neutral primes (Blechert et al., 2014)

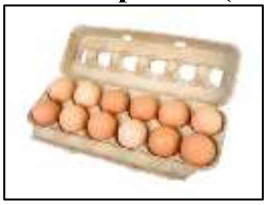
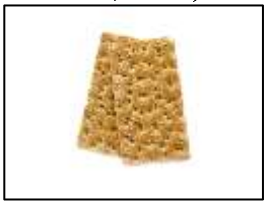
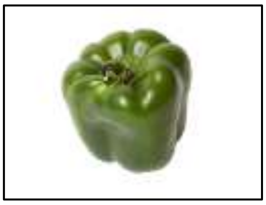

Negative Primes

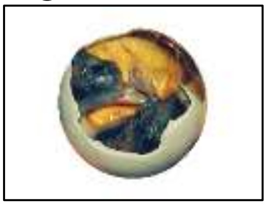
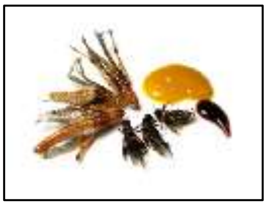
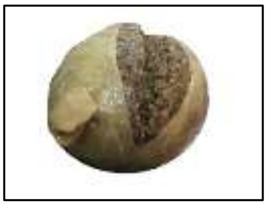

Targets: Valenced nouns (Võ et al., 2009)

Positive nouns		Negative nouns	
WEISHEIT	HELFER	VERLUST	LEICHE
LOBLIED	BRILLANZ	TRAUER	DROHUNG
HEILUNG	CHANCE	ERZFEIND	PANIK
LÄCHELN	ERFOLG	SCHEUSAL	MORDFALL
HOFFNUNG	FREUDE	TRAGIK	HENKER
FESTTAG	GESCHENK	DROHUNG	UNFALL
KLUGHEIT	RETTER	UNMENSCH	BLUTTAT
LACHEN	TRIUMPH	VERRAT	SKLAVE
TALENT	GEWINN	STRAFTAT	GEWALT
WAHRHEIT	FREIHEIT	GALGEN	HORROR

Note: Words other than nouns were changed into the corresponding noun, e.g. "(to) help (German: HELFEN)" was changed into "helper (German: HELFER)", English translation, positive words: wisdom, song of praise, healing, smile, hope, holiday, wisdom, laughter, talent, truth, helper, brilliance, chance, success, joy, gift, savior, triumph, gain, freedom; negative words: loss, mourning, arch-enemy, monster, tragedy, threat, inhumanity, betrayal, crime, gallows, corpse, threat, panic, murder, executioner, accident, bloody deed, slave, violence, horror

Table 5

Means and tests of mean differences for normative data on prime and target stimuli in Study 3.

		Positive stimuli		Neutral stimuli		Negative stimuli				
		M	*SD*	*M*	*SD*	*M*	*SD*	*t*	*df*	*p*
Prime pictures	Palatability	73.00	5.89	46.70	2.60	---	---	7.07	4	.002
	Recogniza-bility	98.80	0.75	99.77	0.25	---	---	2.10	4	.103
Target words	Emotional valence	2.27	0.26	---	---	-2.31	0.20	62.16	38	< .001

Note: Ratings for prime pictures on a scale from 0 to 100 (Blechert et al., 2014); ratings for target words on a scale from -3 to 3 (Võ et al., 2009).

1.2) Indecisiveness Scale (Frost & Shows, 1993), German translation

Cf. Study 2a

1.3) Hunger questionnaire

Instructions:
Please answer these questions based on your current condition.

Bitte beantworten Sie diese Fragen auf Ihren momentanen Zustand bezogen.

Items:

- How hungry are you right now?
- How much appetite do you have right now?
- How much desire do you feel to eat right now?

[1 = *not at all to* 7 = *very much*]

- *Wie hungrig sind Sie gerade?*
- *Wie viel Appetit haben Sie gerade?*
- *Wie viel Lust verspüren Sie gerade, etwas zu essen?*

[1 = gar nicht to 7 = sehr]

1.4) Valence ratings

Instructions:
Please answer some questions about the food shown.
Please refer to your own personal, subjective assessment in the following questions.

Bitte beantworten Sie einige Fragen zu dem abgebildeten Lebensmittel.
Beziehen Sie sich bei den folgenden Fragen auf Ihre ganz persönliche, subjektive Einschätzung.

Items:

- How much would you personally like to eat this food? [1 = *not at all* to 9 = *very much*]
- How attractive do you personally find this food? [1 = *extremely unattractive* to 9 = *extremely attractive*]
- How do you personally rate this food? [1 = *extremely negative* to 9 = *extremely positive*]

- *Wie gern würden Sie persönlich dieses Lebensmittel essen? [1 = äußerst ungern to 9 = äußerst gern]*
- *Wie attraktiv finden Sie persönlich dieses Lebensmittel? [1 = äußerst unattraktiv to 9 = äußerst attraktiv]*
- *Wie bewerten Sie persönlich dieses Lebensmittel? [1 = äußerst negativ to 9 = äußerst positiv]*

1.5) Recognizability

Instructions:
Please answer the following questions:

Bitte beatworten Sie noch folgende Fragen:

Items:

- Have you seen this food in the reaction time task? [*yes; no; uncertain*]
- How well recognisable do you find the food? [1 = *not at all* to 7 = *very much*]
- How familiar are you with the food? [1 = *not at all* to 7 = *very much*]

- Haben Sie dieses Lebensmittel in der Reaktionszeitaufgabe gesehen? [*ja*; *nein*; *unsicher*]
- Wie gut erkennbar finden Sie das Lebensmittel? [1 = *gar nicht* to 7 = *sehr*]
- Wie vertraut sind Sie mit dem Lebensmittel? [1 = *gar nicht* to 7 = *sehr*]

2) Additional analyses for Study 3

In order to eliminate the possibility of an existing effect having remained undetected, we conducted additional analyses. First, we considered a correlation between indecisiveness and the strength of the evaluative priming effect for negative primes only. There was no significant correlation between the strength of the priming effect for negative primes and indecisiveness, $r = -.06$, $p = .59$. Further, as the priming effect tends to increase during the course of the whole procedure (Herring et al., 2013), it is possible that the correlation with indecisiveness differed between blocks. Thus, we tested for a correlation between the complete priming effect and indecisiveness for each block separately, which did not emerge however, all $|r| < .1$, ns.
In addition to the reaction times we analyzed error rates. Just like with the reaction times we subtracted the error rates in congruent trials from those in incongruent trials, resulting in an index in which higher values mirrored a stronger priming effect. We did this across positive and negative primes, as well as for positive and negative primes separately, and for the individual favorite prime. However, the results have to be interpreted with caution, as only 4.5% of trials contained errors. The correlation coefficients can be viewed in Table 6. Indecisiveness only correlated with the error-based priming effect index of the favorite prime. Contrary to our hypothesis, the correlation was positive, meaning that indecisive participants exhibited – at least for their favorite food– a stronger evaluative priming effect than their decisive counterparts.
As in the preceding studies, we reran the main analyses using the IS-Short and IS-AI. We multiplied *p*-values by 2 to account for the additional tests. Results replicated the findings from the main analysis, with the priming effect neither correlating with the IS-Short $\tau = -.02$, $p = .78$, nor with the IS-AI, $\tau = -.04$, $p = .65$. Non-parametrical correlations were used to due to deviations from normality.

Table 6

Correlation coefficients for the correlation between indecisiveness and priming effects (error rates) in Study 3.

	Indec.	TPE	PPE	FPE	NPE
Indec.	-				
TPE	-.09	-			
PPE	.13	.46**	-		
FPE	.26**	.26*	.43**	-	
NPE	-.17	.68**	.07	.09	-

Notes: Indec. = indecisiveness, TPE = total priming effect, PPE = priming effect for positive primes, FPE = priming effect for favorite prime, NE = priming effect for negative primes; all analyses are based on Kendall's tau (τ) due to deviations from normal distribution; due to the exploratory nature of the analyses all *p*-values have been adjusted using the Bonferroni-correction for the number of correlations calculated with indecisiveness (4).

*$p < .05$ **$p < .001$

Item-Total-Correlation (ESM 4)

Table 7

Corrected item-total-correlations of the Indecisiveness Scale items across all studies.

Item	Text	Study 1	Study 2a	Study 2b	Study 3
1	I try to put off making decisions.	0.68	0.59	0.66	0.61
2 (r)	I always know exactly what I want.	0.60	0.66	0.63	0.57
3 (r)	I find it easy to make decisions.	0.69	0.69	0.66	0.73
4	I have a hard time planning my free time.	0.57	0.48	0.48	0.41
5 (r)	I like to be in a position to make decisions.	0.56	0.57	0.53	0.53
6 (r)	Once I make a decision, I feel fairly confident that it is a good one.	0.70	0.55	0.59	0.63
7	When ordering from a menu, I usually find it difficult to decide what to get.	0.55	0.44	0.48	0.31
8 (r)	I usually make decisions quickly.	0.63	0.49	0.46	0.64
9 (r)	Once I make a decision, I stop worrying about it.	0.48	0.50	0.47	0.61
10	I become anxious when making a decision.	0.72	0.60	0.60	0.54
11	I often worry about making the wrong decision.	0.65	0.61	0.63	0.66
12	After I have chosen or decided something, I often believe I've made the wrong choice or decision.	0.62	0.60	0.52	0.69
13	I do not get assignments done on time because I cannot decide what to do first.	0.52	0.49	0.50	0.43
14	I have trouble completing assignments because I can't prioritize what is most important.	0.56	0.46	0.46	0.38
15	It seems that deciding on the most trivial thing takes me a long time.	0.66	0.72	0.63	0.68

Notes: (r) = reversely coded; item 2 (marked) reflects evaluation difficulty. The high item-total-correlation suggests that indecisive individuals also embrace evaluation difficulties. Correlations are corrected in that the respective item is excluded from the scale to prevent inflated correlations.

Distribution of Indecisiveness Scores (ESM 5)

Table 8

Descriptive statistics of studies using the Indecisiveness Scale in samples preselected for clinical symptoms and percentage of participants above respective clinical sample mean in Studies 1 – 3.

Source	Diagnostic category	*N*	*M (SD)*		% above clinical sample *M* Study 1	Study 2a	Study 2b	Study 3
Crone et al. (2010)	HD	156	41.6 (10.5)		35.8	41.8	44.5	43.7
Grisham et al. (2010)	HD	23	48.6 (12.7)		11.9	19.4	19.4	22.5
	MD/AD	17	44.4 (8.1)		24.2	31.8	30.8	32.5
	HC	20	39.7 (9.1)		--	--	--	--
Hall et al. (2013)	HD	363	24.0 (7.3)[a]		38.4	31.3	39.8	30.0
	HC	85	18.7 (6.0)[a]		--	--	--	--
Sip et al. (2016)	OCD	18	50.1 (n/s)		9.9	13.9	14.7	20.0
	HC	16	33.3 (n/s)		--	--	--	--
Steketee et al. (2003)	HD	95	50.1 (12.5)		9.9	13.9	14.7	20.0
	OCD	21	40.6 (10.0)		38.4	43.8	47.4	46.2
	HC	40	35.5 (7.7)		--	--	--	--
Tolin & Villavicencio (2011)	HD	42	29.4 (7.4)[a]		9.3	11.9	17.1	11.2
	OCD	29	23.5 (9.3)[a]		38.4	31.3	47.4	30.0
	HC	36	13.81 (5.5)[a]		--	--	--	--
				N	151	201	211	80
				IS-15: *M (SD)*	37.0 (11.2)	40.2 (10.5)	41.0 (9.5)	41.2 (10.3)
				IS-AI: *M (SD)*	21.6 (7.3)	21.7 (6.7)	23.9 (6.2)	22.1 (6.3)

Notes: HD = hoarding disorder; MD = mood disorder; AD = anxiety disorder; OCD = obsessive compulsive disorder; HC = healthy controls; IS-15 = Indecisiveness Scale score based on all 15 items; IS-AI = Indecisiveness Scale score based on 9 negative worded items ("Aversive Indecisiveness"). Sum scores were used for comparability because most studies report Indecisiveness Scale sum scores.

[a] Values only given for subscale based on 9 negative worded items ("Aversive Indecisiveness")

Histograms of Indecisiveness Scale sum scores per study

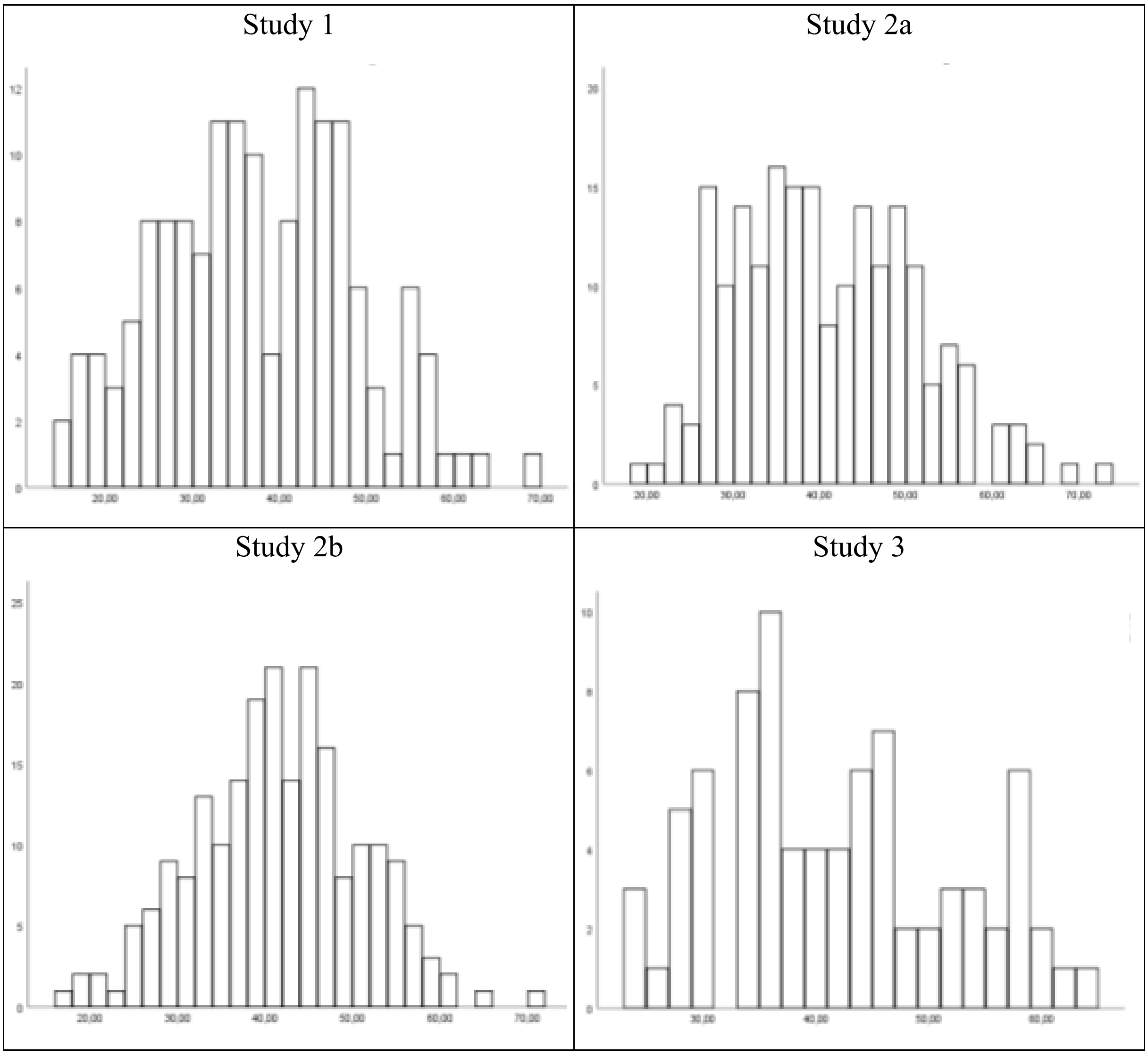

References

Blechert, J., Meule, A., Busch, N. A. & Ohla, K. (2014). Food-pics: An image data-base for experimental research on eating and appetite. *Frontiers in Psychology, 5*, 1-10. doi: 10.3389/fpsyg.2014.00617

Cloutier, J., Heatherton, T. F., Whalen, P. & Kelley, W. M. (2008). Are attractive people rewarding? Sex differences in the neural substrates of facial attractiveness. *Journal of Cognitive Neuroscience, 20*, 941-951. doi:10.1162/jocn.2008.20062

Corneille, O., Monin, B., & Pleyers, G. (2005). Is positivity a cue or a response option? Warm glow vs. evaluative matching in the familiarity for attractive and not-so-attractive face. *Journal of Experimental Social Psychology*, *41*(4), 431-437.

Crone, C., Kwok, C., Chau, V., & Norberg, M. M. (2019). Applying attachment theory to indecisiveness in hoarding disorder. *Psychiatry Research, 273,* 318–324.

DeBruine, L. M. (2004). Facial resemblance increases the attractiveness of same-sex faces more than other-sex faces. *Proceedings of the Royal Society of London B: Biological Sciences*, *271*, 2085-2090.

Frost, R. O., & Shows, D. L. (1993). The nature and measurement of compulsive indecisiveness. *Behaviour Research and Therapy, 31*(7), 683–692.

Grisham, J. R., Norberg, M. M., Williams, A. D., Certoma, S. P., & Kadib, R. (2010). Categorization and cognitive deficits in compulsive hoarding. *Behaviour Research and Therapy, 48*(9), 866–872.

Hall, B. J., Tolin, D. F., Frost, R. O., & Steketee, G. (2013). An exploration of comorbid symptoms and clinical correlates of clinically significant hoarding symptoms: Research Article: Patterns of comorbidity among people with hoarding behavior. *Depression and Anxiety, 30*(1), 67–76.

Herring, D. R., White, K. R., Jabeen, L. N., Hinojos, M., Terrazas, G., Reyes, S. M., ... Crites, S. J. (2013). On the automatic activation of attitudes: A quarter century of evaluative priming research. *Psychological Bulletin, 139*, 1062-1089.

Lauderdale, S. A., & Oakes, K. (2021). Factor Structure of the Revised Indecisiveness Scale and Association with Risks for and Symptoms of Anxiety, Depression, and Attentional Control. *Journal of Rational-Emotive & Cognitive-Behavior Therapy*, *39*(2), 256–284.

Rassin, E., Muris, P., Franken, I., Smit, M., & Wong, M. (2007). Measuring general indecisiveness. *Journal of Psychopathology and Behavioral Assessment, 29*(1), 60–67.

Sip, K. E., Muratore, A. F., & Stern, E. R. (2016). Effects of context on risk taking and decision times in obsessive-compulsive disorder. *Journal of Psychiatric Research*, *75*, 82–90.

Steketee, G., Frost, R. O., & Kyrios, M. (2003). Cognitive Aspects of Compulsive Hoarding. *Cognitive Therapy and Research*, 17.

Tolin, D. F., & Villavicencio, A. (2011). An exploration of economic reasoning in hoarding disorder patients. *Behaviour Research and Therapy, 49*(12), 914–919.

Võ, M. L.-H., Conrad, M., Kuchinke, L., Hartfeld, K., Hofmann, M. F. & Jacobs, A. M. (2009). The Berlin Affective Word List Reloaded (BAWL-R). *Behavior Research Methods, 41*, 534-538.

Zebrowitz, L. A. & Rhodes, G. (2002). Nature let a hundred flowers bloom: The multiple ways and wherefores of attractiveness. In G. Rhodes & L. Zebrowitz (Hrsg.), *Facial attractiveness: Evolutionary, cognitive, and social perspectives* (S. 262-293). Westport, US: Ablex Publishing.
